# The Minnesota attributable risk of kidney donation (MARKD) study: a retrospective cohort study of long-term (> 50 year) outcomes after kidney donation compared to well-matched healthy controls

**DOI:** 10.1186/s12882-023-03149-7

**Published:** 2023-05-01

**Authors:** David M. Vock, Erika S. Helgeson, Aidan F. Mullan, Naim S. Issa, Sujana Sanka, Alison C. Saiki, Kristin Mathson, Alanna M. Chamberlain, Andrew D. Rule, Arthur J. Matas

**Affiliations:** 1 Division of Biostatistics, School of Public Health, University of Minnesota, 2221 University Ave SE, Room 200, Minneapolis, MN 55414 USA; 2 Department of Quantitative Health Sciences, Mayo Clinic, Rochester, MN USA; 3 Division of Nephrology and Hypertension, Department of Medicine, Mayo Clinic, Rochester, MN USA; 4 Surgery Clinical Trials Office, Department of Surgery, University of Minnesota, Minneapolis, MN USA; 5 Department of Cardiovascular Medicine, Mayo Clinic, Rochester, MN USA; 6 Division of Transplantation, Department of Surgery, University of Minnesota, Minneapolis, MN USA

**Keywords:** Cohort studies, Follow-up studies, Kidney transplantation, Living donors, Nephrectomy

## Abstract

**Background:**

There is uncertainty about the long-term risks of living kidney donation. Well-designed studies with controls well-matched on risk factors for kidney disease are needed to understand the attributable risks of kidney donation.

**Methods:**

The goal of the Minnesota Attributable Risk of Kidney Donation (MARKD) study is to compare the long-term (> 50 years) outcomes of living donors (LDs) to contemporary and geographically similar controls that are well-matched on health status. University of Minnesota (n = 4022; 1st transplant: 1963) and Mayo Clinic LDs (n = 3035; 1st transplant: 1963) will be matched to Rochester Epidemiology Project (REP) controls (approximately 4 controls to 1 donor) on the basis of age, sex, and race/ethnicity. The REP controls are a well-defined population, with detailed medical record data linked between all providers in Olmsted and surrounding counties, that come from the same geographic region and era (early 1960s to present) as the donors. Controls will be carefully selected to have health status acceptable for donation on the index date (date their matched donor donated). Further refinement of the control group will include confirmed kidney health (e.g., normal serum creatinine and/or no proteinuria) and matching (on index date) of body mass index, smoking history, family history of chronic kidney disease, and blood pressure. Outcomes will be ascertained from national registries (National Death Index and United States Renal Data System) and a new survey administered to both donors and controls; the data will be supplemented by prior surveys and medical record review of donors and REP controls. The outcomes to be compared are all-cause mortality, end-stage kidney disease, cardiovascular disease and mortality, estimated glomerular filtration rate (eGFR) trajectory and chronic kidney disease, pregnancy risks, and development of diseases that frequently lead to chronic kidney disease (e.g. hypertension, diabetes, and obesity). We will additionally evaluate whether the risk of donation differs based on baseline characteristics.

**Discussion:**

Our study will provide a comprehensive assessment of long-term living donor risk to inform candidate living donors, and to inform the follow-up and care of current living donors.

**Supplementary Information:**

The online version contains supplementary material available at 10.1186/s12882-023-03149-7.

## Background

A living donor kidney transplant is the best treatment option for a patient with end-stage kidney disease (ESKD) as it provides better patient and graft survival and quality of life than either dialysis or a deceased donor transplant [1–13]. However, living donors (LDs) must undergo a major operative procedure associated with morbidity and mortality and the potential for adverse long-term consequences of living with a single kidney [14–58]. Practice guidelines, including those by the Kidney Disease: Improving Global Outcomes (KDIGO) consortium, allow a donor’s glomerular filtration rate (GFR) threshold to be as low as 60 ml/min/1.73m^2^ as an acceptance criterion for donation[59, 60]. The British Transplant Society 2018 guidelines and the European Renal Best Practice 2013 guidelines even allow GFR below 60 ml/min/1.73m^2^ in older donors[61, 62]. Since postdonation GFR is about 65% of the predonation value due to hypertrophy of the retained kidney, many LDs will have a GFR less than 60 ml/min/1.73m^2^ early after nephrectomy. For others, a decrease of GFR with aging or new onset disease will subsequently result in GFR less than 60 ml/min/1.73m^2^. Numerous studies in the general population have shown that individuals with GFR of less than 60 ml/min/1.73m^2^ have increased long-term risks including continuing loss of kidney function and higher rates of cardiovascular disease (CVD) and mortality [63–87]. Those observations may not be relevant to LDs who have an abrupt, isolated reduction in GFR in the absence of any underlying disease process or comorbid illness [88–93].

### Existing studies of attributable long-term risk of kidney donation

Several studies have sought to estimate the attributable risk of donor nephrectomy on mortality, ESKD, CVD, and other outcomes. Single-center and registry studies comparing LDs to the general population have not shown increased long-term CVD, ESKD, or mortality risk [14, 44–54]. Indeed, studies of longitudinal renal function in LDs have shown a small annual increase in GFR or estimated GFR (eGFR) for more than 15 years after donation [94–98]. A number of studies with average follow-up of less than 10 years compared LDs to matched healthy controls and found no difference in CVD and mortality [52–54, 58]. However, a 2014 Norwegian study with a median follow-up of 18 years found increased CVD and mortality in LDs compared to healthy controls; the authors attributed the difference in their findings compared to prior ones to longer follow-up [55]. A subsequent study based on the same population reported an increased risk of ischemic heart disease in LDs [99]. Two studies – the 2014 study from the Norwegian group, the other from the United States (US) - reported an increased risk of ESKD among LDs [55, 56]. Studies of postdonation pregnancies, compared to the general population, have generally not identified an increased risk of adverse fetal or neonatal outcomes. However, two studies comparing postdonation to predonation pregnancies reported an increased risk of hypertensive complications in the postdonation pregnancies, and one study reported an increased risk of hypertensive complications compared to healthy selected non-donors [100–102].

### Limitations of prior work

In a review of studies reporting increased long-term risk for LDs, Janki et al. identified that both the Norwegian study reporting an increased risk of CVD, mortality, and ESKD, and the US study reporting increased risk of ESKD, selected non-donor control populations that were healthier than the donors, resulting in an over estimation of risk [103]. In addition, the definition of ESKD differed between donors and non-donors in the US study, potentially biasing the results. A meta-analysis of the cardiac studies concluded that there was no increase in CVD disease or mortality in LDs [104]. For the two studies identifying an increased risk of ESKD among LDs, the number of events was small, and there have been additional concerns about methodology, including: controls were non-contemporaneous and from a different geographic location; lack of renal function assessment for controls at the date corresponding to the date of donation; and concerns about the choice of statistical methods [105–115].

A key limitation in existing studies is that there was no determination of family history of chronic kidney disease (CKD) or ESKD for controls. In the Norwegian study, all 9 donors who developed ESKD were first-degree relatives of the recipient; in the US study, 83 of the 99 who developed ESKD were related to the recipient. Numerous studies in the general population have shown that individuals related to a person with ESKD have an increased risk of ESKD [116–122]. Wainright et al., using the Organ Procurement and Transplantation Network (OPTN) database, reported that LDs who were first-degree relatives of the recipient had significantly increased risk of ESKD (highest in identical twins) compared to LDs who were distant relatives or were unrelated to the recipient [123].

There have only been a few studies of pregnancy risk postdonation [100–102, 124, 125]. Studies have been limited by low numbers of events, and the design has generally been restricted to a before-and-after study design which may result in an over estimation of risk.

Finally, to date, most studies of LDs matched to healthy controls had limited follow-up. In the US study by Muzaale et al.,[56] the mean follow-up was 7.2 years, and in the Norwegian study, the median follow-up was 15.1 years. Steiner has emphasized that long-term follow-up is necessary to determine if kidney donation is associated with ESKD risk, since most LDs are relatively young at donation and most ESKD develops later in life [126–128]. In addition, the majority of kidney diseases progress slowly, so that short-term LD follow-up will only identify LDs developing rapidly progressive disease (e.g., immunologic). Long-term follow-up is required to determine the impact of diseases associated with aging, specifically hypertension and diabetes mellitus (DM), the two most common causes of ESKD in the US general population and the US LD population [129]. Analyses of US national LD data (20-year follow-up) and University of Minnesota (UMN) LDs have shown that the rate of ESKD increases postdonation; and, specifically, the cumulative incidence of ESKD due to DM or hypertension increases many years after donation [130, 131]. Thus, short-term studies will miss most postdonation ESKD.

The recognition of significant gaps in our understanding of long-term LD outcomes has resulted in calls for more complete long-term LD follow-up studies [132]. In the US, this is reflected in statements from the Advisory Committee on Organ Transplantation[132], The Organ Donation and Recovery Improvement Act (ODRIA (P.L. 108–216) (Sect. 7) and (Sec 371 A)[133], and by the Health Resources and Services Administration in testimony to Congress[134] and on its website [135]. Consensus conferences have argued for continued, systematic collection and reporting of long-term LD outcomes [136, 137]; reasons included: (a) LDs and their potential recipients need accurate outcome information for informed consent, and (b) donation is a public trust, and the community has an obligation to continue to collect detailed information on outcomes. And, importantly, LDs themselves are asking for this information [138–141]. Of concern, between 2004 and 2019 (because of the pandemic the number of LDs decreased in 2020 and 2021) the number of LDs has only increased 3%[129]; it has been suggested that the uncertainty regarding LD risk may be partly responsible.

To address these calls to action and limitations of existing studies, the Minnesota Attributable Risk of Kidney Donation (MARKD) study will compare the long-term risks for LDs from UMN and Mayo Clinic (first donation in 1963 and up to 60 years of follow-up) to the long-term risks for contemporaneous, geographically similar, healthy matched controls from the Rochester Epidemiology Project (REP). By reviewing medical records, we additionally plan to identify a subset of controls from the REP with known normal kidney function at a date corresponding to the date of donation and known family history of CKD and be able to match controls to LDs on these important risk factors. Consequently, we will provide the best data to date to inform prospective candidates of the long-term risks of kidney donation.

## Methods

### Overview of study design

**Fig. 1:**
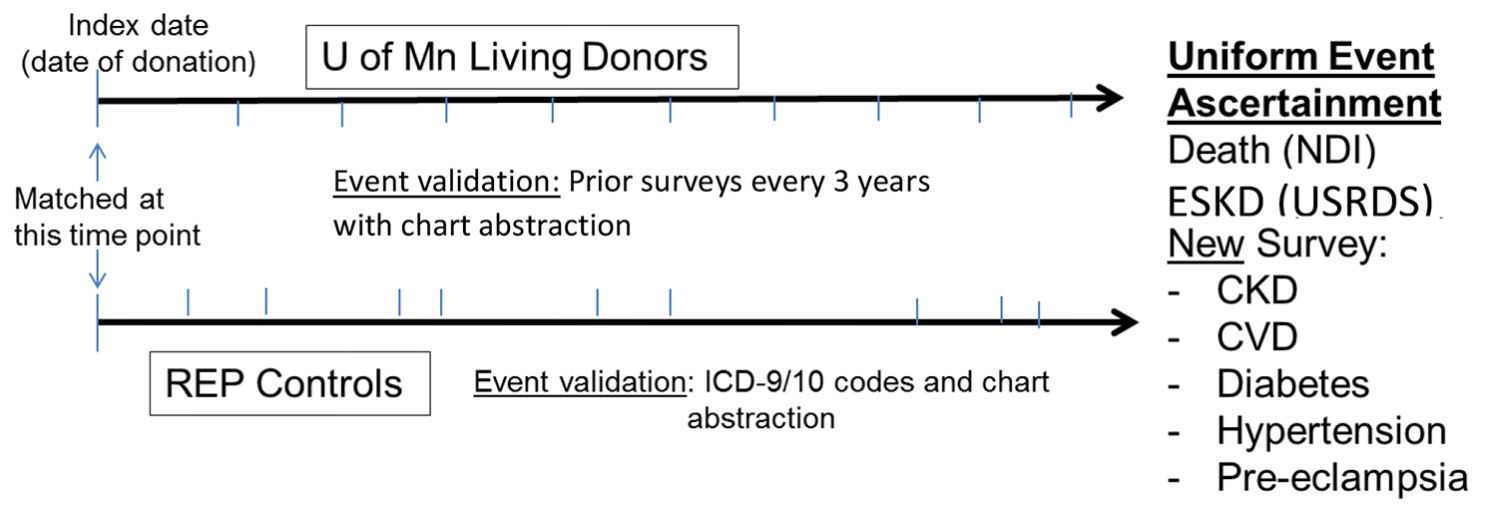
Matched Cohort Study Design

This is a matched cohort study of LDs from UMN and Mayo Clinic with contemporaneous, chart-validated, healthy matched REP controls with an index date (date of donation for LD) between 1963 and 2012. This time frame was chosen since the focus of the study is on long-term (> 10-year postdonation) outcomes (see Fig. 1). New outcome data will be ascertained from national registries, including the National Death Index (NDI, mortality), United States Renal Data System (USRDS, ESKD), and a new survey of health outcomes. The new data will be augmented with information collected from prior surveys (UMN LDs) and medical record review (UMN LDs and REP controls). Study criteria for donors includes: 1) provided research authorization and 2), US residency. Known non-US residents will be excluded from donor and control cohorts since national database information is unavailable for these individuals.

### Aims and hypotheses

The MARKD study aims to determine long-term attributable LD risk by comparing well-matched controls with LDs. LDs will be compared with matched controls to determine: (a) whether LDs are at greater risk for the rare outcomes of mortality and ESKD; (b) whether LDs are at increased risk for CVD, cardiovascular mortality, and/or increased risk of intermediate measures of kidney disease (proteinuria, CKD [eGFR < 45 and eGFR < 30 ml/min/1.73m^2^ ]); (c) whether the trajectory of eGFR differs between LDs and controls; d) whether there are differences in the above outcomes for those developing de novo kidney disease and/or disease that might affect the kidneys (e.g., hypertension, DM, proteinuria); and e) whether there are differences in pregnancy outcomes (e.g., preeclampsia) between LDs and controls. The overall hypothesis is that LDs will have relatively small, slightly increased, but clinically insignificant, differences in the incidence of ESKD, mortality, intermediate endpoints, and pregnancy complications compared to well-matched control groups.

### Study cohorts

#### UMN LD cohort

From January 1, 1963 through December 31, 2012, there were 4022 LDs at UMN who met the study criteria. Most LDs were from the upper Midwest region of the US, and detailed descriptions of the UMN LD cohort have been published [45, 50]. At donation, laboratory and demographic variables, surgical information, and peri-operative course information is collected. Prior to 2003, LDs were surveyed intermittently. In 2003, all donors were contacted to be surveyed and asked to consent to be surveyed every three years. Since 2003, consenting LDs are surveyed postdonation at regular intervals (6, 12, and 24 months, and then every 3 years). Those not returning to the clinic are sent a survey (by mail; or, if preferred, by email) to determine health status. If the survey is not returned, the LD is contacted by phone monthly, for 3 months. At least one phone call is made in the evening and one on weekends to increase response rates. LDs are also asked to provide alternate contacts. For LDs who become lost to follow-up UMN uses these contacts to ask for a current phone number and address. Available internet search engines and other databases (e.g., Accurint.com, Peoplefinder.com) are also used to identify LDs lost to follow-up.

At each contact, LDs are asked about hypertension, DM, and other diagnoses requiring treatment. Female donors are asked about pregnancy history. LDs are also asked to provide recent lab test results and copies of medical records; alternatively, with LDs’ permission, UMN investigators contact local clinics for recent medical records that include clinical notes and laboratory test results, including serum creatinine, glucose, urinalysis, and urinary protein measurements. Those without recent information are asked to get a history, physical, and labs at their local physician’s office and have the results sent to UMN. For LDs willing to do the tests but without insurance coverage, UMN pays for them. A summary of the clinical and demographic characteristics of the UMN LD cohort is given in Table 1.

**Table 1:** Characteristics of the University of Minnesota (UMN) and Mayo Clinic donors

	UMN donors (N = 4022)	Mayo donors (N = 3035)
Years from donation to last survey or History & Physical (mean ± SD)	22.04 ± 11.93	
No returned surveys	9.5%	
Sex (%)		
Female	2294 (57.0%)	1668 (55.0%)
Male	1728 (43.0%)	1354 (44.6%)
Unknown	0 (0%)	13 (0.4%)
Age at donation (mean ± SD)	39.02 ± 11.64	42.52 ± 11.98
Unknown	0 (0%)	13 (0.4%)
Race (%)		
Asian	40 ( 1.0%)	31 (1.0%)
Black or African American	95 ( 2.4%)	50 (1.6%)
Caucasian / White	3796 (94.4%)	2840 (93.6%)
Hawaiian Or Other Pacific Islander	1 ( 0.0%)	4 (0.1%)
Multi-racial	90 ( 2.2%)	26 (0.9%)
Unknown	0 (0%)	84 (2.8%)
Ethnicity (%)		
Hispanic/Latino	99 ( 2.5%)	66 (2.2%)
Non-Hispanic/Non-Latino	3428 (85.2%)	2600 (85.7%)
Unknown	495 (12.3%)	369 (12.2%)
Index decade (%)		
1960s	99 ( 2.5%)	45 (1.5%)
1970s	658 (16.4%)	271 (8.9%)
1980s	796 (19.8%)	218 (7.2%)
1990s	1040 (25.9%)	454 (15.0%)
2000s	1200 (29.8%)	1647 (54.3%)
2010–2012	229 ( 5.7%)	400 (13.2%)
Donor relationship (%)		
Child	474 (11.8%)	493 (16.2%)
Parent	932 (23.2)	422 (13.9%)
Full Sibling	1579 (39.3%)	934 (30.8%)
Half Sibling	21 ( 0.5%)	25 (0.8%)
Identical Twin	15 ( 0.4%)	5 (0.2%)
Not Related	562 (14.0%)	662 (21.8%)
Other Relative	241 ( 6.0%)	156 (5.1%)
Spouse	198 ( 4.9%)	327 (10.8%)
Unknown	0 (0%)	11 (0.4%)
Creatinine at donation (mean ± SD)	0.91 ± 0.16	0.90 ± 0.19
Unknown	0.7%	1.1%
BMI at donation (mean ± SD)	25.88 ± 4.36	27.38 ± 5.11
Unknown	1.5%	0.5%
Glucose at donation (mean ± SD)	93.46 ± 14.77	96.00 ± 14.12
Unknown	4.9%	1.0%
DBP at donation (mean ± SD)	73.26 ± 9.92	73.16 ± 12.22
Unknown	1.9%	4.8%
SBP at donation (mean ± SD)	119.85 ± 13.11	120.62 ± 15.22
Unknown	1.9%	4.8%

#### Mayo clinic LD cohort

Between 1963 and December 31, 2012, 3035 LD transplants were performed who met the study criteria, with most LDs from the upper Midwest region of the US. The acquisition and storage of all relevant clinical data for all LDs are currently available via the Transplant Center clinical databases, which interfaces with the institution’s electronic medical record to access laboratory and demographic variables, surgical information, and peri-operative course. The Mayo Clinic LD program provides an additional source of long-term LD follow-up that will: (a) increase the sample size to increase the statistical power for analyses of certain outcomes (ESKD and mortality) and (b) add a second site to assess the generalizability of findings. A summary of the clinical and demographic characteristics of the Mayo LD cohort is given in Table 1.

#### Rochester epidemiology project

The development and use of the REP have been described in detail [142–145]. In brief, the REP is a medical records linkage system that allows detailed collection of data on a well-defined population (Olmsted and surrounding Counties, Minnesota, US) starting in the early 1960s. The REP was developed with the recognition that nearly all residents of Olmsted County received their medical care at one of two hospitals and/or at affiliated institutions because of the central location of the hospitals in an area in which there are no other competing medical centers. In addition, other small private practices have partnered with the major medical centers in the area to contribute to the REP. As such, population estimates for the Olmsted County by REP are slightly higher and more comprehensive than those reported in the US Census (1970, 104.1%; 1980, 103.5%; 1990, 102.4%; and 2000, 102.7% of the US Census counts) [144]. The characteristics of the population are similar to those of residents from the Upper Midwest of the US [145, 146]. The records contain details of every inpatient hospitalization at the affiliated hospitals, every outpatient visit to the office, clinic, or emergency department, as well as every laboratory result and correspondence concerning each patient. The medical records are easily retrievable because the REP has maintained extensive indices based on clinical and histologic diagnoses (i.e., diagnosis codes) and surgical procedures. The result is the linkage of medical records from nearly all sources of medical care utilized by the Olmsted County population. Each year, more than half of the population is examined at one of the REP facilities, and about 85–100%, depending on the age group, have at least one REP contact over a 3-year period [142].

The REP represents the same geographical region as both the UMN and Mayo LDs with the same time frame starting in the 1960s, making it an ideal control group (reference cohort) for long-term follow-up.

### Control matching algorithm, validation, and abstraction

Our goal is to identify, for each LD, individuals in the REP database who would have been eligible for kidney donation on the same date as the LD based on the information in the medical record.

*Pilot studies*:

As we were unsure of the most efficient way to use diagnostic codes and chart abstraction for excluding controls that would not qualify as donors, we performed two pilot studies to optimize our selection of exclusionary codes and exclusionary criteria on chart abstraction.

First, we identified all approximately 180,000 individuals who have not opted out of the use of their medical records for research (per Minnesota statute) in the REP Olmsted County cohort with a potential index date within the age and time period window of the donors. Within the REP, code sets have been developed for 355 different specific diseases using International Classification of Disease 9th and 10th revisions (ICD-9, ICD-10), Hospital Adaptation of the International Classification of Diseases (HICDA), and Berkson codes (a local coding system that predates ICD-9 codes). On review of these code sets, we determined that 173 of these were conditions/diseases that if truly present, would always lead to exclusion as a donor. Of these exclusionary diseases, 26 were prevalent on or prior to the index date of at least 1,000 individuals within the REP. However, we did not want to exclude too many of our potential controls that were false positives for these exclusionary diseases. Thus, we (Drs. Matas and Issa) performed a manual chart review on 10 random individuals for each of these disease code sets. We found that some codes within these code sets corresponded to conditions that were true contraindications for donation (e.g., kidney disease), but some were for mild disease or other conditions which were not absolute contraindications (e.g., urinary incontinence that was mild, depression that was mild and transient, arrhythmias – palpitation with negative cardiac workups). The individual codes for these 26 code sets were refined to better correspond to conditions that would be absolute contraindications for donation. Subsequently, all 173 exclusionary diseases (including the 26 that were adjusted to be made more specific for exclusion and less sensitive) were used to exclude individuals from being matched as controls. See **Supplemental Materials** for a full list of exclusionary conditions.

Second, after excluding individuals with these 173 diseases, we performed a pilot study of 117 randomly sampled individuals to abstract in detail their medical status, available data at or prior to the potential index date and during follow-up, and any potential concerns with their candidacy as a kidney donor on the index date. From this pilot study, we identified 5 exclusionary conditions (psychiatric conditions, substance abuse, hypertension, abnormal serum creatinine or urine protein, and obesity) that were relatively common and missed by the exclusionary code set. Psychiatric conditions, substance abuse, and hypertension are common and require medical record review as many are mild and do not warrant exclusion. Abnormal serum creatinine, urine protein, or body mass index (BMI) are simply underdiagnosed and, thus, require medical record review to be detected for exclusion.

#### Initial matching algorithm

**Fig. 2:**
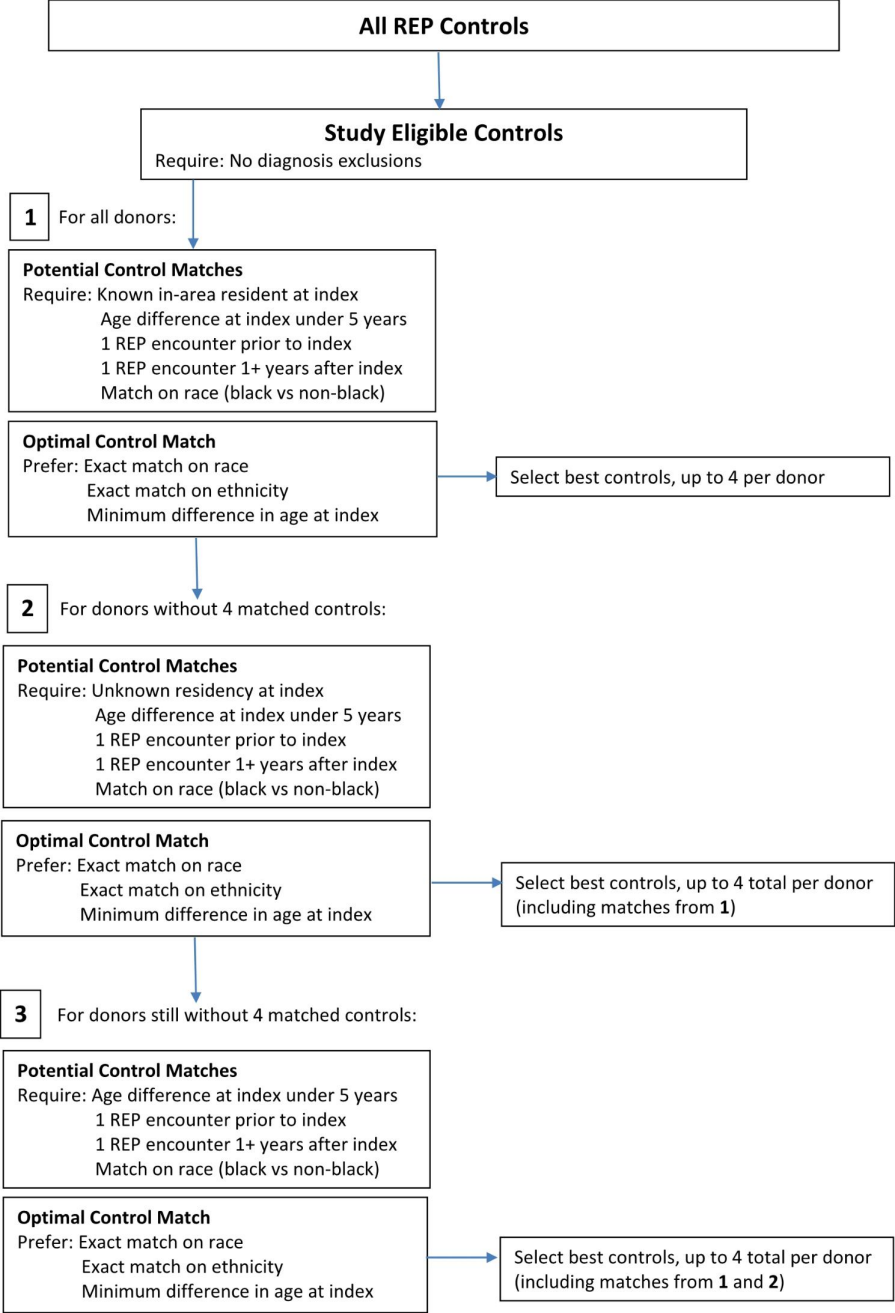
REP Control Match Flow Chart

After finalizing the list of exclusionary codes, up to 4 potential controls were identified for every donor. All controls were residents of Olmsted County (or surrounding counties) at some point and lacked any exclusionary diagnoses before the index date (date of donation for the matched LD) or up to 1 year after the index date. The 1-year window after the index date was used because some diseases may be present on the index date but not diagnosed due to the intermittent nature of clinic visits in the medical record. This matching was accomplished first for the UMN donors and then for the Mayo Clinic donors. These potential REP controls were initially identified based on the following requirements: (1) Olmsted County or surrounding county resident at index date, (2) at least one REP encounter prior to index and at least one REP encounter one year or more after index, (3) age difference between LD and potential REP control at index less than 5 years, and (4) exact match on broad racial categories (Black vs. non-Black). If more than four controls meet these criteria, the four best controls based on (1) exact match on more granular racial categories (White or unknown, Black, Asian, Hawaiian/Pacific Islander, Other), (2) exact match on ethnicity (Hispanic, non-Hispanic), and (3) minimum difference in age at index was selected. If fewer than four controls were identified, the matching criteria were broadened to allow for unknown county of residence at the index date and subsequently known non-resident of Olmsted or surrounding counties at the index date. The full algorithm is provided in Fig. 2. From this initial algorithmic matching over 16,000 and 14,000 potential controls were identified for the UMN and Mayo Clinic donors, respectively.

#### Control validation and medical record abstraction

Subsequently, all identified potential control charts (paper and electronic) are being manually reviewed and abstracted into a REDCap database by the abstraction team, which consists of a nurse practitioner who previously worked in a kidney transplant clinic, nurses, and medical research trainees. Abstraction guidelines with screenshots of the database are maintained and updated as needed for use by all abstractors. After orientation is completed, each abstractor completes a set of 20 cases to ensure consistency with these guidelines. Chart abstraction involves two steps: (1) validation of the potential control (i.e., ensuring that the subject would have been a suitable donor at the index date), and then, if valid: (2) capturing clinical and laboratory data. The validation step involves reviewing medical records prior to the index date for contraindications for kidney donation. The following conditions are often found by abstractors resulting in exclusion of possible control: hypertension (blood pressure over 140/90 mm Hg), Proteinuria (1 + dipstick, 24-hour urine protein > 200 mg, protein osmolarity ratio > 0.42), impaired kidney function (creatinine > 1.3 mg/dL in men, > 1.1 mg/dL in women), non-tobacco substance abuse (interferes with lifestyle or requires hospitalization/treatment), obesity (BMI > 40 kg/m^2^), history of recent cancer (other than non-melanoma skin cancers), severe depression/anxiety (untreated and interferes with lifestyle), history of urological abnormalities (significant structural/functional abnormalities of the urinary tract), heart disease, liver disease, and chronic obstructive pulmonary disease.

Similar to the donor candidate assessment, certain conditions are allowed for controls at the time of index if they are otherwise healthy. A BMI of 35–40 kg/m^2^ is allowed if the control is deemed to be in otherwise very good health as BMIs in this range are allowed in some donors. As with donors, medically well-controlled hypertension is allowed in controls over 50 years of age. Controls on more than 1 non-diuretic antihypertensive medication or more than 1 diuretic antihypertensive medication are excluded. Depression/anxiety is allowed if well controlled, infrequent, and acute episodes had occurred in the distant past relative to the index date. Substance abuse is also allowed if use was in the remote past; current marijuana and alcohol use is allowed as long as it does not interfere with work and social function. Patients who were pregnant at the index date or within 3 months postpartum, or hospitalized within 2 weeks of index date (for a non-exclusionary reason), may be rematched to a different donor at a different index date.

If it is unclear if a certain control would have been eligible to donate given their medical history, a review by kidney donation experts (Drs. Matas, Rule, and Issa) is used to approve or exclude the potential control. The abstraction guidelines are subsequently updated to ensure future consistency. In addition, the abstractor team meets monthly after completing a reliability chart (same chart for each abstractor) to ensure similar abstractions among the abstractor team.

If a control is validated, abstractors proceed to collect additional baseline and follow-up data. This includes any mention of family history of DM, hypertension, CKD, or ESKD. We obtain the closest laboratory data measures before and after the index date for baseline. These measures include: height, weight, blood pressure (avoiding blood pressure obtained during visits to the emergency department), serum creatinine, blood urea nitrogen (BUN), serum glucose, urine glucose, and any assessment of proteinuria/albuminuria (avoiding those with concurrent urinary tract infections).

### Outcomes

Outcomes include mortality, CVD and cardiovascular mortality, serial creatinine (to determine eGFR trajectory and chronic kidney disease) and other laboratory measures, pregnancy outcomes and complications, and development of diseases that frequently lead to CKD (e.g., hypertension, DM, and obesity). Outcomes will be ascertained from national registries, medical record review of donors and REP controls, and a new survey administered to both donors and controls. A list of data collected, data source, and frequency of data collection is provided in Table 2 and is summarized below.

**Table 2:** Data collection sources and frequency for REP controls and UMN donors. CR: Chart Review, DS: Donor survey + chart review if available (every 3 years), SS: Study Survey, LD: Lab data (every year)

	Source of data	
Data	UMN Donors	REP Controls
**Baseline characteristics**		
Demographics	CR	CR^1^
History of substance abuse^2^	CR	CR
Diagnosis of Depression/Anxiety Disorder^3^	CR	CR
High cholesterol	CR, SS	CR, SS
Hypertension^4^	CR, SS	CR, SS
Family history of kidney disease, DM, HTN	CR, DS, SS	CR, SS
Smoking history	CR, DS, SS	CR, SS
**Lab measurements**	Requested every 3 years with study survey	Recorded every year if available
BUN/Urea	CR	CR, LD
Glucose	CR	CR, LD
Urine protein^5^	CR	CR, LD
BMI	CR, DS, SS	CR, LD, SS
Creatinine	CR, DS, SS	CR, LD, SS
Blood pressure	CR, DS, SS	CR, LD, SS
**Outcomes**		
CVD^6^	DS, SS	CR, SS
Diabetes	DS, SS	CR, SS
Cancer	DS, SS	CR, SS
Hematuria	DS, SS	CR, SS
High cholesterol treated with medication	DS, SS	CR, SS
Hypertension treated with medication	DS, SS	CR, SS
Pregnancy history^7^	DS, SS	CR, SS
Proteinuria	DS, SS	SS
Kidney disease/ abnormal kidney function/ ESKD	DS, SS, USRDS	CR, SS, USRDS
Vital status and cause of death	DS, SS, NDI	CR^8^, SS, NDI

^1^ Chart review for controls includes abstraction of data from electronic transfer from the Rochester Epidemiology Project (REP) database, Mayo clinic paper records, Olmstead Medical Center electronic and paper record, Olmstead Community Hospital electronic and paper records, Epic, and the REP browser (electronic record system) from date of first REP encounter to date of last follow-up

^2^Permissible substance abuse at donation/donation is defined as a remote history of marijuana use with no active use around the time of the donation date or current marijuana use as long as it does not interfere with lifestyle. Remote other substance use is not a contraindication to donation as long as its truly remote

^3^Permissible depression/anxiety at donation/donation is defined as well controlled, in the distant past prior to the donation date, or involving few episodes of care

^4^Permissable hypertension at donation/donation includes that which is well controlled (< 140/90), treated with a diuretic antihypetensive agent, a non-diuretic antihypertensive agent, or both (1 diuretic and 1 non-diuretic hypertensive agent), age needs to be > 50 years. Exclude if age < 50 years or not well controlled or using 2 or more non-diuretic agents or using 2 or more diuretic agents

^5^Urine Dipstick, 24 H Urine Protein, Urinalysis Predicted 24-Hour Protein, Protein-Osmolality Ratio, Urine Protein to Creatinine Ratio, Urine Albumin to Creatine Ratio, 24 H Urine Albumin

^6^Congestive heart disease, myocardial infarction, cerebrovascular accident, transient ischemic attack, angioplasty, stents

^7^Number and year of pregnancies, pregnancy outcome (singleton, twins, triplets, ≥ 4, abortion, miscarriage, fetal death), gestational length (full term, pre-term), and pregnancy complications (gestational hypertension, gestational diabetes, proteinuria, eclampsia, preeclampsia)

^8^Proof of life noted for medical encounters with no data

#### National registries: mortality and ESKD

To determine mortality/causes of death and ESKD, patient identifiers (name, birthdate, social security number, address) for the UMN and Mayo donors and the REP controls will be sent in a secure manner to the NDI and to the USRDS, respectively.

#### Medical record review: mortality, ESKD, and other outcomes

Once a control is validated, as noted above, abstractors collect follow-up data from the medical record. Lab measures obtained after index are limited to up to one value per calendar year and include: weight, blood pressure, serum creatinine, serum glucose, BUN, serum creatinine, and urine protein. For efficiency of abstraction, the database is prefilled with all electronically available values from the medical record and then validated by the abstractor. Finally, clinical diagnoses and the year they occurred are recorded from the medical record.

As described above, when UMN LDs are surveyed, they are also asked to provide recent lab test results and copies of medical records. At the time of survey administration, abstractors collect follow-up data from medical record of the UMN health system and affiliated clinics. With LDs’ permission, UMN investigators will contact other clinics for recent medical records that include clinical notes and laboratory test results.

#### Survey data: mortality, ESKD, and other outcomes

UMN donors and validated REP controls not known to be deceased will be sent a standardized survey to determine outcomes. This survey is based on the existing survey that has been used for UMN donors, but modified to be usable with both LDs and controls. This survey supplements the capture of key data that may not be consistently documented in the medical record with specific items including: hematuria or proteinuria of at least 6 months duration, hypertension, DM, heart failure, myocardial infarction, angioplasty or stent, angina, smoking history, and pregnancy history; and family history of hypertension, DM, or ESKD (see **Supplemental Materials** for the survey). We will additionally capture cancer information to use as a negative control (see statistical analysis section).

The survey will allow for uniform ascertainment of outcomes between UMN donors and REP controls. Because Mayo LDs have not been routinely contacted about health outcomes after donation, they do not have a similar long-term follow-up history as UMN LDs from which to validate survey responses. Because of this and budgetary constraints, only the REP controls and UMN donors will be surveyed.

UMN donors will be contacted to complete the survey using a similar process as described previously for the 3-year postdonation follow-up surveys. Data obtained from this new survey will be used to supplement data collected in the triannual surveys sent to UMN donors previously.

For REP controls, mailing addresses and phone numbers will be obtained from recent Olmsted County medical care episodes. REP controls without a recent medical visit will be queried in Accurint™ to determine if they are alive and if so, identify their most recent mailing address and phone number. Up to three mailed surveys, with the first including an incentive item for participation, will be sent to the controls followed by up to 5 phone calls (including evenings and weekends). After surveying the first validated control for each REP donors, we will prioritize surveying controls for donors who lack completed surveys by a matched control.

### Database security, quality assurance, and ethics

We have established a secure database to combine data from each source. The same combination of manual review, quality control reports, and built-in logic checks that has been used to ensure data quality for UMN LD survey responses will be used for all surveys in this study to ensure the integrity of the data. This study has been approved by the Institutional Review Boards of the University of Minnesota and the Mayo Clinic.

### Statistical considerations

#### Primary control group and matching algorithms

Although the REP control matching algorithm outlined in Sect. 2.4 matches on age at index date and race/ethnicity, there may be important differences between the LDs and potential REP controls on other characteristics. We plan to rematch LDs to these potential REP controls based on other clinical characteristics. Although laboratory data is nearly complete in the LDs (due to the extensive donor evaluation), these data are less complete among otherwise healthy potential controls. Indeed, it is possible that measurement of some characteristics (e.g., serum creatinine), particularly among young, otherwise healthy individuals, may indicate suspicion of disease. Thus, requiring potential controls to have complete laboratory data may lead to selection bias. However, including potential controls with missing laboratory values may lead to detection bias as some of these controls may have an unrecognized underlying disease. We will consider several different sets of characteristics to match on to create control cohorts which vary between minimizing detection bias and minimizing selection bias. This will allow us to determine the variability/range of incidence rates for each outcome that are dependent on how the controls are matched to donors. A prior study assessing risk of CKD with kidney stones had this same challenge of missing serum creatinine levels in the REP, but found reasonably consistent findings in analyses that minimized detection bias versus analyses that minimized selection bias [147].

For the **primary analysis**, we consider controls for whom we can ascertain BMI, systolic and diastolic blood pressure, use of anti-hypertensive medications, smoking history, and known normal serum creatinine, normal blood urea nitrogen (particularly relevant in the 1960s where serum creatinine testing may not have been done), or normal urine protein (with no abnormal values for any of these 3 tests) identified within 10 years prior to the index date (minimize detection bias as we avoid any controls who may have had unrecognized kidney disease due to absence of kidney function testing). We additionally will attempt to include family history of CKD at the index date (either through chart review or from survey results) as a factor in the matching. Urine protein tests allowed are comprehensive (urine dipstick protein, 24 h urine protein, 24 h urine albumin, urine protein to creatinine ratio, urine protein to osmolality ratio, and urine albumin to creatinine ratio). However, we expect most will be urine dipstick proteins. Normal is defined using the reference range at the time of the test. Our preliminary data suggested that younger controls will often not have a baseline serum creatinine level, though about half will have a urine protein assessment. Since most kidney disease in younger adults is proteinuric, a normal urine protein test in younger adults (age < 50 years) will be considered sufficient for inclusion in this analysis.

We plan to use optimal, sparse 1:k matching with refined covariate balance[148, 149] to ensure that the patients who are in each group (donors and controls) are as similar as possible based on age, sex, race/ethnicity, BMI, systolic and diastolic blood pressure, hypertension, smoking history, and family history of CKD. Briefly, this method allows one to exactly match on a small number of covariates (in this sense the method is sparse because it does not consider all possible matched pairs). The matching algorithm allows the near fine balance on discrete covariates between the intervention and control group (this means the distribution of the covariate is the same in the two groups but individuals may be matched to others with different levels of the covariate, i.e., frequency matching). An advantage of this approach is that it allows prioritization for balance on certain covariates over others. Finally, individuals are matched to minimize the overall difference between individuals using Mahalanobis distance or difference in propensity score (in this sense the match is optimal). The number of matched controls will depend on the number of potential controls with the available data. For each cohort of matched controls, we will assess the quality of matches by 1) computing the standardized difference of means for each covariate, as well as two-way interactions and squares and 2) for continuous covariates, examining quantile–quantile (QQ) plots, which compare the empirical distributions of each variable in the donor and control groups. Covariates for which there is an imbalance (e.g., standardized mean difference greater than 0.1) may be discretized and matched either exactly or to achieve near fine balance. Because matching is part of the design phase (as opposed to the analysis phase), we can repeat the matching several times until an acceptable balance is achieved without inflating type I error rates. If not enough controls with a matching characteristic (e.g., family history of CKD) are identified for matching to LDs, then the presence of the characteristics in both LDs and controls will be corrected for with multivariable adjustment for the analyses in predicting outcomes. Given that causal analysis is a rapidly developing field we will additionally consider state-of-the-art methodologies for determining optimal matches as new discoveries are made in this field.

#### Control groups for sensitivity analysis

In addition to the set of controls selected for the primary analysis, we will consider additional broader groups from the REP from which to match. As in the primary control group, we plan to use sparse optimal 1:k matching to match REP patients in these groups to LDs.

i) REP patients for whom we can ascertain BMI, systolic and diastolic blood pressure, diagnosis of hypertension, smoking history, and normal renal function (normal serum creatinine, normal blood urea nitrogen, or normal urine protein). This group does not consider family history of CKD.
ii) REP patients for whom we can ascertain BMI, systolic and diastolic blood pressure, diagnosis of hypertension, and smoking history with no known abnormal renal function (abnormal serum creatinine, abnormal blood urea nitrogen, or abnormal urine protein). This group does not consider family history of CKD or require renal function testing to have been done.
iii) A group that does not consider family history of CKD, require renal function testing to have been done, or consider clinical characteristics (however, patients with known contraindications to donation would have already been excluded and those with known abnormal renal function will still be excluded).
iv) Additionally, we will construct control groups using matching algorithms as close as possible to those used in previous studies[55, 56] to assess the effect of different matching criteria on the rates of each outcome.

For each control group (primary and sensitivity cohorts) we match to LDs, we will estimate the incidence rate of each outcome.

#### Statistical analyses details

Log-rank tests and Kaplan Meier curves will be used as preliminary assessments to compare the risk of all-cause mortality and cardiovascular mortality, CVD, ESKD, CKD (eGFR < 45; eGFR < 30 ml/min/1.73m^2^), proteinuria, DM, and hypertension after index date between LDs and healthy controls. Our study is powered based on these analyses. Cox proportional hazards models will also be used to test for an increased risk of adverse outcomes in living LDs versus controls while directly accounting for the variables used for matching (to account for any possible residual imbalance between the cohorts similar to how one may adjust for characteristics in a randomized controlled trial to account for residual imbalance and to improve efficiency). In analyses involving Mayo LDs and their associated controls we will account for donation center (Mayo vs. UMN) as a covariate in the model. If the proportional hazards assumption fails, an alternative method such as an accelerated failure time model will be considered. Indeed, the risk of mortality and ESKD may disproportionately increase with long-term follow-up on the 60-year scale of this study [123, 130, 131]. A similar approach will be used to test for increased risk of outcomes for LDs versus controls after development of DM, hypertension, proteinuria, de novo kidney disease, or pregnancies with gestational hypertension or preeclampsia, but additional measures (e.g. matching, adjustment) will be used to account for the time of disease onset.

In order to evaluate biases in the selection of controls and ascertain outcomes, we will compare the risk of non-kidney-related cancer between donors and controls using the previously described methods. As cancer risk is lower in donors than the general population, we expect a similar lower risk in controls [150]. If controls are well matched, we do not expect the risk of non-kidney related cancer to differ between donors and controls. If a difference in cancer risk is identified, it may indicate imbalance in our matching and it will be further investigated using methods described in Shi et al. [151].

We will also evaluate if the effect of donation differs across baseline characteristics (i.e. evaluate if a characteristic modifies the effect of donation) by testing the significance of the interaction term between donation and the characteristic in the model. These models will also allow us to assess the attributable risk of donation in LDs with certain characteristics at donation. We will specifically assess if age, BMI, smoking, donation eGFR, and race/ethnicity modify the effect of donation on ESKD and mortality. As a sensitivity analysis, the analyses will be repeated using a subset of REP controls having at least 5 years of medical history preceding the index date.

Logistic regression models fit using generalized estimating equations will be used to test for differences in the odds of gestational hypertension and preeclampsia between LDs and controls for all pregnancies occurring after the index date, while accounting for women having potentially multiple pregnancies. Adjustment variables to be considered in the model include: race/ethnicity, age at pregnancy, gravidity, and previous occurrence of pregnancy complications. Secondary analyses using logistic regression models will test for differences in the odds of these complications in the first pregnancy after the index date, adjusting for having previous pregnancies, and second pregnancy after the index date, adjusting for the occurrence of pregnancy complications in the first post-index date pregnancy.

A linear mixed effects model will be used to test the difference in the trajectory of eGFR over time between living LDs and healthy controls. This model will initially have a linear effect for time with subject-specific random intercepts and slopes, but natural splines will be used to investigate nonlinear temporal trajectories.

#### Power

Given the results identified using preliminary matching algorithms and chart review we assume that we will be able to identify 3 controls for 98% of LDs and at least one of those controls will be included in a more restrictive matched subset (i.e., known presence of CKD, known serum creatinine/urine protein at or prior to index date). For the following power analyses, the type-1 error probability is set to 5%.

For estimating the minimal detectible hazard ratio for all-cause mortality, we note that at present, 12% of UMN LDs, 7% of all Mayo LDs, and 11% of identified preliminary controls are deceased. With a mortality rate of 11% in our study population, we estimate that our minimal detectible hazard ratio with 80% power will be 1.13 for a 3:1 matching and 1.16 for a 1:1 matching.

In UMN LDs, approximately 13.5% have developed eGFR < 45 ml/min/1.73m^2^ during follow-up. In our analysis comparing the risk of developing an eGFR < 45 between UMN LDs and healthy controls, we conservatively estimate that eGFR < 45 will develop in approximately 8 to 14% of our study population. With this assumption, our minimal detectable hazard ratio will be between 1.20 and 1.15 for a 3:1 matching and between 1.25 and 1.18 for a 1:1 matching.

Among the 301 UMN LDs who developed DM after donation without first developing CVD, 57 individuals (19%) later developed CVD. Assuming identification of a 1:1 match for approximately 80% (240) of these donors and that the overall proportion of individuals in our study population who develop CVD after DM ranges from 15 to 23%, we will have 80% power to detect a hazard ratio between 1.94 and 1.70.

## Discussion

The MARKD study will evaluate the attributable long-term risk of kidney donation by matching LDs from two transplant centers with controls from a large, contemporaneous, geographically similar epidemiological cohort. A similar study with the number of LDs and healthy matched controls and length of detailed follow-up could not be done anywhere else worldwide. While our geographic location is constrained in terms of ethnic/racial diversity, no other transplant centers have detailed long-term donor follow-up with a truly well-matched control group. The Norwegian study described previously has the longest healthy matched-control study to date [55]. However, it has a much smaller sample size and numerous limitations as previously described [103–115]. In the US, the OPTN did not start collecting Social Security numbers of donors until 1994, limiting linkages to other databases to obtain information on ESKD and mortality. Also, before 1996, LDs with ESKD listed with the OPTN for a transplant were not necessarily identified as a LD [152]. And today, even 6-month follow-up in the OPTN database remains poor [152–155].

The MARKD study will focus on LDs in two LD programs that started in 1963, and REP-controls dating back to 1963 with comprehensive medical records that are ongoing. A unique aspect of our study is the granular long-term follow-up. The UMN LD program has surveyed LDs every 3 years. The REP links the full life course medical records of nearly all Olmsted County providers, allowing electronic (ICD-9/10 codes) and manual (chart abstraction) review of the comprehensive medical record of controls. No other study of this size provides: (a) this length of follow-up (more than three decades longer follow-up than the US OPTN registry), and (b) detailed ongoing follow-up of LDs and healthy-matched cohorts. Developing a matched control cohort from the REP will not only allow us to provide the best and the longest data on long-term LD outcomes to date, but will also lay the groundwork for future analyses.

A major innovative aspect of our study is our contemporaneous healthy matched control cohort. Unlike prior studies, we will more clearly delineate matched controls that reflect the health status of donors just prior to the date of donation. This will be accomplished by identifying controls known to be healthy and with normal renal function at the index date (matched to donation date) who are from the same geographical region of residence (Upper Midwest) as donors, and by obtaining granular data on controls to allow for optimal matching of controls to LDs on key characteristics (including family history of CKD).

Longitudinal follow-up of both LDs and REP controls provides detailed, uniquely available health information on events preceding ESKD and mortality. In contrast to previous studies, we will compare LDs and healthy-matched REP controls on the much more common intermediate endpoints (e.g., low GFR; CVD) that occur before ESKD or death. Additionally, we will be able to study outcomes of LDs who develop new comorbidities late after donation (e.g., obesity, hypertension, DM, early or late GFR < 45 ml/min/1.73m^2^, proteinuria). This will provide information about whether the impact of these comorbidities differs for those with 1 vs. 2 kidneys. That is, we will be able to assess if differences in long-term outcomes between donors and controls are mediated by differences in postdonation comorbidities.

We will be among the first studies to evaluate modifiers of the effect of donation on ESKD and mortality. There have been several studies of risk factors for developing ESKD in the donor population. However, these identified risk factors are also risk factors for ESKD in the general population. By using well-matched REP controls, we will be able to assess the effect of donation for “ideal” LDs and for LDs who, at donation, have risk factors for CKD. That is, ours will be among the first studies to test if the attributable risk of an outcome with kidney donation is modified by clinical and demographic factors (e.g., age, BMI, smoking, donation eGFR).

Another strength of our study is uniform event ascertainment. Use of national databases (NDI, USRDS), access to long-term historical data, and a new survey sent at the same time to LDs and controls will allow us to ascertain events by the same approach in both LDs and controls. Simultaneously obtaining outcomes for both groups the same way will avoid a differential ascertainment bias. Further, the granular long-term follow-up of REP controls and the UMN LD program will provide independent sources for validating the timing and onset of self-reported outcomes on the new survey. Thus, we will be able to provide the most complete and detailed information on long-term LD outcomes as compared to a well-matched healthy control cohort.

### Dissemination

Findings from this study will be disseminated through peer-reviewed journals, conference presentations, and targeted dissemination/presentation to advocacy and stakeholder groups. To translate our findings into an understandable format for LDs and their care team we will develop a user-friendly long-term risk calculator. This freely available, interactive online calculator or app will allow candidate donors (and their providers) to estimate their risk of each outcome (including intermediate outcomes) at different time intervals with versus without kidney donation.

### Summary

The MARKD study will provide the best data, to date, on the attributable risk of kidney donation. The data will inform prospective candidates of the long-term risks (if any) of kidney donation; and inform best practices in long-term clinical follow-up, as well as the design of long-term health maintenance/surveillance of donors.

## Electronic supplementary material

Below is the link to the electronic supplementary material.

Supplementary Material 1

Supplementary Material 2

Supplementary Material 3

## Data Availability

Data sharing is not applicable to this article as no datasets were generated or analyzed during the current study.

## Abbreviations

BMI: body mass index
BUN: blood urea nitrogen
CKD: chronic kidney disease
CVD: cardiovascular disease
DBP: diastolic blood pressure
DM: diabetes mellitus
eGFR: estimated glomerular filtration rate
ESKD: end-stage kidney disease
GFR: glomerular filtration rate
HICDA: Hospital Adaptation of the International Classification of Diseases
ICD-9: International Classification of Diseases, 9th revision
ICD-10: International Classification of Diseases, 10th revision
KDIGO: Kidney Disease: Improving Global Outcomes
LD: living donor
MARKD: Minnesota Attributable Risk of Kidney Donation
NDI: National Death Index
OPTN: Organ Procurement and Transplantation Network
QQ: quantile–quantile
REP: Rochester Epidemiology Project
SBP: systolic blood pressure
UMN: University of Minnesota
US: United States
USRDS: United States Renal Data System

## References

[1] Wolfe RA, Ashby VB, Milford EL, Ojo AO, Ettenger RE, Agodoa LY, et al. Comparison of mortality in all patients on dialysis, patients on dialysis awaiting transplantation, and recipients of a first cadaveric transplant. N Engl J Med. 1999 Dec;341(2):1725–30. 10.1056/NEJM199912023412303 10580071

[2] Evans RW, Manninen DL, Garrison LP, Hart LG, Blagg CR, Gutman RA, et al. The quality of life of patients with end-stage renal disease. N Engl J Med. 1985 Feb;28(9):553–9. 10.1056/NEJM198502283120905 3918267

[3] Schnuelle P, Lorenz D, Trede M, Van Der Woude FJ. Impact of renal cadaveric transplantation on survival in end-stage renal failure: evidence for reduced mortality risk compared with hemodialysis during long-term follow-up. J Am Soc Nephrol. 1998 Nov;9(11):2135–41. 10.1681/ASN.V9112135 9808102

[4] Cosio FG, Alamir A, Yim S, Pesavento TE, Falkenhain ME, Henry ML, et al. Patient survival after renal transplantation: I. The impact of dialysis pre-transplant. Kidney Int. 1998 Mar;53(3):767–72. 10.1046/j.1523-1755.1998.00787.x 9507225

[5] Meier-Kriesche HU, Port FK, Ojo AO, Rudich SM, Hanson JA, Cibrik DM, et al. Effect of waiting time on renal transplant outcome. Kidney Int. 2000 Sep;58(3):1311–7. 10.1046/j.1523-1755.2000.00287.x 10972695

[6] Meier-Kriesche HU, Kaplan B. Waiting time on dialysis as the strongest modifiable risk factor for renal transplant outcomes: a paired donor kidney analysis.Transplantation. 2002 Nov27;74(10):1377–81. 10.1097/00007890-200211270-00005 12451234

[7] Gill JS, Tonelli M, Johnson N, Pereira BJG. Why do preemptive kidney transplant recipients have an allograft survival advantage? Transplantation. 2004 Sep 27;78(6):873–9. 10.1097/01.tp.0000130204.80781.68 15385807

[8] Mange KC, Joffe MM, Feldman HI. Effect of the use or nonuse of long-term dialysis on the subsequent survival of renal transplants from living donors. N Engl J Med. 2001 Mar;8(10):726–31. 10.1056/NEJM200103083441004 11236776

[9] Papalois VE, Moss A, Gillingham KJ, Sutherland DE, Matas AJ, Humar A. Pre-emptive transplants for patients with renal failure: an argument against waiting until dialysis.Transplantation. 2000 Aug27;70(4):625–31. 10.1097/00007890-200008270-00016 10972221

[10] Mange KC, Weir MR. Preemptive renal transplantation: why not? Am J Transplant. 2003 Nov;3(11):1336–40. 10.1046/j.1600-6143.2003.00232.x 14525592

[11] Innocenti GR, Wadei HM, Prieto M, Dean PG, Ramos EJ, Textor S et al. Preemptive living donor kidney transplantation: do the benefits extend to all recipients? Transplantation. 2007 Jan 27;83(2):144–9. 10.1097/01.tp.0000250555.46539.65 17264810

[12] Becker BN, Rush SH, Dykstra DM, Becker YT, Port FK. Preemptive transplantation for patients with diabetes-related kidney disease. Arch Intern Med. 2006 Jan;9(1):44–8. 10.1001/archinte.166.1.44 16401809

[13] Abecassis M, Bartlett ST, Collins AJ, Davis CL, Delmonico FL, Friedewald JJ et al. Kidney transplantation as primary therapy for end-stage renal disease: a National Kidney Foundation/Kidney Disease Outcomes Quality Initiative (NKF/KDOQITM) conference. Clin J Am Soc Nephrol. 2008 Mar;3(2):471–80. 10.2215/CJN.05021107 PMC2390948 18256371

[14] Najarian JS, Chavers BM, McHugh LE, Matas AJ. 20 years or more of follow-up of living kidney donors.Lancet. 1992 Oct3;340(8823):807–10. 10.1016/0140-6736(92)92683-7 1357243

[15] Bay WH, Hebert LA. The living donor in kidney transplantation. Ann Intern Med. 1987 May;106(5):719–27. 10.7326/0003-4819-106-5-719 3551714

[16] Matas AJ, Bartlett ST, Leichtman AB, Delmonico FL. Morbidity and mortality after living kidney donation, 1999–2001: survey of United States transplant centers. Am J Transplant. 2003 Jul;3(7):830–4. 12814474

[17] Friedman AL, Peters TG, Jones KW, Boulware LE, Ratner LE. Fatal and nonfatal hemorrhagic complications of living kidney donation. Ann Surg. 2006 Jan;243(1):126–30. 10.1097/01.sla.0000193841.43474.ec PMC1449959 16371747

[18] Johnson EM, Remucal MJ, Gillingham KJ, Dahms RA, Najarian JS, Matas AJ. Complications and risks of living donor nephrectomy.Transplantation. 1997 Oct27;64(8):1124–8. 10.1097/00007890-199710270-00007 9355827

[19] Penn I, Halgrimson CG, Ogden D, Starzl TE. Use of living donors in kidney transplantation in man. Arch Surg. 1970 Aug;101(2):226–31. 10.1001/archsurg.1970.01340260130021 PMC3003931 4916014

[20] Davison JM, Uldall PR, Walls J. Renal function studies after nephrectomy in renal donors. Br Med J. 1976 May;1(6017):1050–2. 10.1136/bmj.1.6017.1050 PMC1639921 1268548

[21] Ringdén O, Friman L, Lundgren G, Magnusson G. Living related kidney donors: complications and long-term renal function. Transplantation. 1978 Apr;25(4):221–3. 10.1097/00007890-197804000-00013 635986

[22] Vincenti F, Amend WJ, Kaysen G, Feduska N, Birnbaum J, Duca R, et al. Long-term renal function in kidney donors. Sustained compensatory hyperfiltration with no adverse effects. Transplantation. 1983 Dec;36(6):626–9. 10.1097/00007890-198336060-00006 6659058

[23] Hakim RM, Goldszer RC, Brenner BM. Hypertension and proteinuria: long-term sequelae of uninephrectomy in humans. Kidney Int. 1984 Jun;25(6):930–6. 10.1038/ki.1984.112 6381857

[24] Miller IJ, Suthanthiran M, Riggio RR, Williams JJ, Riehle RA, Vaughan ED, et al. Impact of renal donation. Long-term clinical and biochemical follow-up of living donors in a single center. Am J Med. 1985 Aug;79(2):201–8. 10.1016/0002-9343(85)90010-5 3895908

[25] Tapson JS, Marshall SM, Tisdall SR, Wilkinson R, Ward MK, Kerr DN (1985). Renal function and blood pressure after donor nephrectomy. Proc Eur Dial Transplant Assoc Eur Ren Assoc.

[26] Anderson CF, Velosa JA, Frohnert PP, Torres VE, Offord KP, Vogel JP et al. The risks of unilateral nephrectomy: status of kidney donors 10 to 20 years postoperatively. Mayo Clin Proc. 1985 Jun;60(6):367–74. 10.1016/s0025-6196(12)60845-3 3999807

[27] Bohannon LL, Barry JM, Norman DJ, Bennett WM. Renal function 27 years after unilateral nephrectomy for related donor kidney transplantation. J Urol. 1988 Oct;140(4):810–1. 10.1016/s0022-5347(17)41822-2 3418806

[28] Hoitsma AJ, Paul LC, Van Es LA, Koene RA (1985). Long term follow-up of living kidney donors. A two-centre study. Neth J Med.

[29] Sobh M, Nabeeh A, el-Din AS, el-Housseiny null, Ibrahiem I, el-Kenavy M et al. Long-term follow-up of the remaining kidney in living related kidney donors. Int Urol Nephrol. 1989;21(5):547–53. 10.1007/BF02549594 2613485

[30] Mathillas O, Attman PO, Aurell M, Blohmé I, Brynger H, Granérus G (1985). Proteinuria and renal function in kidney transplant donors 10–18 years after donor uninephrectomy. Ups J Med Sci.

[31] Smith S, Laprad P, Grantham J. Long-term effect of uninephrectomy on serum creatinine concentration and arterial blood pressure. Am J Kidney Dis. 1985 Sep;6(3):143–8. 10.1016/s0272-6386(85)80017-2 4036959

[32] O’Donnell D, Seggie J, Levinson I, Meyers AM, Botha JR, Myburgh JA et al. Renal function after nephrectomy for donor organs. S Afr Med J. 1986 Feb 1;69(3):177–9. 3511548

[33] Talseth T, Fauchald P, Skrede S, Djøseland O, Berg KJ, Stenstrøm J, et al. Long-term blood pressure and renal function in kidney donors. Kidney Int. 1986 May;29(5):1072–6. 10.1038/ki.1986.109 3523003

[34] Williams SL, Oler J, Jorkasky DK. Long-term renal function in kidney donors: a comparison of donors and their siblings. Ann Intern Med. 1986 Jul;105(1):1–8. 10.7326/0003-4819-105-1-1 3521424

[35] Dunn JF, Nylander WA, Richie RE, Johnson HK, MacDonell RC, Sawyers JL. Living related kidney donors. A 14-year experience. Ann Surg. 1986 Jun;203(6):637–43. 10.1097/00000658-198606000-00008 PMC1251194 3521509

[36] Kasiske BL, Ma JZ, Louis TA, Swan SK. Long-term effects of reduced renal mass in humans. Kidney Int. 1995 Sep;48(3):814–9. 10.1038/ki.1995.355 7474669

[37] Gossmann J, Wilhelm A, Kachel HG, Jordan J, Sann U, Geiger H, et al. Long-term consequences of live kidney donation follow-up in 93% of living kidney donors in a single transplant center. Am J Transplant. 2005 Oct;5(10):2417–24. 10.1111/j.1600-6143.2005.01037.x 16162190

[38] Torres VE, Offord KP, Anderson CF, Velosa JA, Frohnert PP, Donadio JV, et al. Blood pressure determinants in living-related renal allograft donors and their recipients. Kidney Int. 1987 Jun;31(6):1383–90. 10.1038/ki.1987.153 3302507

[39] Rizvi SAH, Naqvi SAA, Jawad F, Ahmed E, Asghar A, Zafar MN et al. Living kidney donor follow-up in a dedicated clinic.Transplantation. 2005 May15;79(9):1247–51. 10.1097/01.tp.0000161666.05236.97 15880079

[40] Fehrman-Ekholm I, Dunér F, Brink B, Tydén G, Elinder CG. No evidence of accelerated loss of kidney function in living kidney donors: results from a cross-sectional follow-up.Transplantation. 2001 Aug15;72(3):444–9. 10.1097/00007890-200108150-00015 11502974

[41] Ramcharan T, Matas AJ. Long-term (20–37 years) follow-up of living kidney donors. Am J Transplant. 2002 Nov;2(10):959–64. 10.1034/j.1600-6143.2002.21013.x 12482149

[42] El-Agroudy AE, Sabry AA, Wafa EW, Neamatalla AH, Ismail AM, Mohsen T, et al. Long-term follow-up of living kidney donors: a longitudinal study. BJU Int. 2007 Dec;100(6):1351–5. 10.1111/j.1464-410X.2007.07054.x 17941927

[43] Goldfarb DA, Matin SF, Braun WE, Schreiber MJ, Mastroianni B, Papajcik D, et al. Renal outcome 25 years after donor nephrectomy. J Urol. 2001 Dec;166(6):2043–7. 11696703

[44] Fehrman-Ekholm I, Elinder CG, Stenbeck M, Tydén G, Groth CG. Kidney donors live longer.Transplantation. 1997 Oct15;64(7):976–8. 10.1097/00007890-199710150-00007 9381544

[45] Ibrahim HN, Foley R, Tan L, Rogers T, Bailey RF, Guo H, et al. Long-term consequences of kidney donation. N Engl J Med. 2009 Jan;29(5):459–69. 10.1056/NEJMoa0804883 PMC3559132 19179315

[46] Okamoto M, Akioka K, Nobori S, Ushigome H, Kozaki K, Kaihara S, et al. Short- and long-term donor outcomes after kidney donation: analysis of 601 cases over a 35-year period at japanese single center. Transplantation. 2009 Feb;15(3):419–23. 10.1097/TP.0b013e318192dc95 19202449

[47] Fehrman-Ekholm I, Nordén G, Lennerling A, Rizell M, Mjörnstedt L, Wramner L et al. Incidence of end-stage renal disease among live kidney donors.Transplantation. 2006 Dec27;82(12):1646–8. 10.1097/01.tp.0000250728.73268.e3 17198252

[48] Lentine KL, Schnitzler MA, Xiao H, Saab G, Salvalaggio PR, Axelrod D, et al. Racial variation in medical outcomes among living kidney donors. N Engl J Med. 2010 Aug;19(8):724–32. 10.1056/NEJMoa1000950 PMC3041966 20818874

[49] Fournier C, Pallet N, Cherqaoui Z, Pucheu S, Kreis H, Méjean A, et al. Very long-term follow-up of living kidney donors. Transpl Int. 2012 Apr;25(4):385–90. 10.1111/j.1432-2277.2012.01439.x 22356210

[50] Ibrahim HN, Foley RN, Reule SA, Spong R, Kukla A, Issa N, et al. Renal function Profile in white kidney donors: the First 4 decades. J Am Soc Nephrol. 2016 Sep;27(9):2885–93. 10.1681/ASN.2015091018 PMC5004661 26888476

[51] Mjøen G, Reisaeter A, Hallan S, Line PD, Hartmann A, Midtvedt K, et al. Overall and cardiovascular mortality in norwegian kidney donors compared to the background population. Nephrol Dial Transplant. 2012 Jan;27(1):443–7. 10.1093/ndt/gfr303 21636826

[52] Segev DL, Muzaale AD, Caffo BS, Mehta SH, Singer AL, Taranto SE et al. Perioperative mortality and long-term survival following live kidney donation.JAMA. 2010 Mar10;303(10):959–66. 10.1001/jama.2010.237 20215610

[53] Reese PP, Bloom RD, Feldman HI, Rosenbaum P, Wang W, Saynisch P, et al. Mortality and cardiovascular disease among older live kidney donors. Am J Transplant. 2014 Aug;14(8):1853–61. 10.1111/ajt.12822 PMC4105987 25039276

[54] Berger JC, Muzaale AD, James N, Hoque M, Wang JMG, Montgomery RA, et al. Living kidney donors ages 70 and older: recipient and donor outcomes. Clin J Am Soc Nephrol. 2011 Dec;6(12):2887–93. 10.2215/CJN.04160511 PMC3255359 22034505

[55] Mjøen G, Hallan S, Hartmann A, Foss A, Midtvedt K, Øyen O, et al. Long-term risks for kidney donors. Kidney Int. 2014 Jul;86(1):162–7. 10.1038/ki.2013.460 24284516

[56] Muzaale AD, Massie AB, Wang MC, Montgomery RA, McBride MA, Wainright JL, et al. Risk of end-stage renal disease following live kidney donation. JAMA. 2014 Feb;12(6):579–86. 10.1001/jama.2013.285141 PMC4411956 24519297

[57] Weiland D, Sutherland D, Chavers B. Information on 628 living-related kidney donors at a single institution, with long-term follow-up in 472 cases. Transplant Proc. 1984;16:5–7.

[58] Garg AX, Prasad GVR, Thiessen-Philbrook HR, Ping L, Melo M, Gibney EM et al. Cardiovascular disease and hypertension risk in living kidney donors: an analysis of health administrative data in Ontario, Canada.Transplantation. 2008 Aug15;86(3):399–406. 10.1097/TP.0b013e31817ba9e3 18698242

[59] Lentine KL, Kasiske BL, Levey AS, Adams PL, Alberú J, Bakr MA et al. KDIGO Clinical Practice Guideline on the Evaluation and Care of Living Kidney Donors.Transplantation. 2017Aug;101(8S Suppl 1):S1–109. 10.1097/TP.0000000000001769 PMC5540357 28742762

[60] Gaillard F, Legendre C, White CA. GFR Assessment of living kidney donors candidates. Transplantation. 2019 Jun;103(6):1086–93. 10.1097/TP.0000000000002620 30801521

[61] British Transplantation Society. BTS/RA Living Donor Kidney Transplantation Guidelines 2018 [Internet]. 2018. Available from: https://bts.org.uk/wp-content/uploads/2018/07/FINAL_LDKT-guidelines_June-2018.pdf

[62] European Renal Best Practice Transplantation Guideline Development Group. ERBP Guideline on the management and evaluation of the kidney donor and recipient. Nephrol Dial Transplant. 2013 Aug;28(Suppl 2):ii1–71. 10.1093/ndt/gft218 24026881

[63] Damsgaard EM, Frøland A, Jørgensen OD, Mogensen CE. Microalbuminuria as predictor of increased mortality in elderly people. BMJ. 1990 Feb;3(6720):297–300. 10.1136/bmj.300.6720.297 PMC1661920 2106959

[64] Friedman PJ. Serum creatinine: an independent predictor of survival after stroke. J Intern Med. 1991 Feb;229(2):175–9. 10.1111/j.1365-2796.1991.tb00327.x 1997642

[65] Hamdan AD, Pomposelli FB, Gibbons GW, Campbell DR, LoGerfo FW. Renal insufficiency and altered postoperative risk in carotid endarterectomy. J Vasc Surg. 1999 Jun;29(6):1006–11. 10.1016/s0741-5214(99)70241-7 10359934

[66] Matts JP, Karnegis JN, Campos CT, Fitch LL, Johnson JW, Buchwald H. Serum creatinine as an independent predictor of coronary heart disease mortality in normotensive survivors of myocardial infarction. POSCH Group. J Fam Pract. 1993 May;36(5):497–503. 8482933

[67] Dries DL, Exner DV, Domanski MJ, Greenberg B, Stevenson LW. The prognostic implications of renal insufficiency in asymptomatic and symptomatic patients with left ventricular systolic dysfunction. J Am Coll Cardiol. 2000 Mar 1;35(3):681–9. 10.1016/s0735-1097(99)00608-7 10716471

[68] Anderson RJ, O’Brien M, MaWhinney S, VillaNueva CB, Moritz TE, Sethi GK, et al. Mild renal failure is associated with adverse outcome after cardiac valve surgery. Am J Kidney Dis. 2000 Jun;35(6):1127–34. 10.1016/s0272-6386(00)70050-3 10845827

[69] Weiner DE, Tighiouart H, Stark PC, Amin MG, MacLeod B, Griffith JL, et al. Kidney disease as a risk factor for recurrent cardiovascular disease and mortality. Am J Kidney Dis. 2004 Aug;44(2):198–206. 10.1053/j.ajkd.2004.04.024 15264177

[70] Anavekar NS, McMurray JJV, Velazquez EJ, Solomon SD, Kober L, Rouleau JL, et al. Relation between renal dysfunction and cardiovascular outcomes after myocardial infarction. N Engl J Med. 2004 Sep;23(13):1285–95. 10.1056/NEJMoa041365 15385655

[71] Gueyffier F, Boissel JP, Pocock S, Boutitie F, Coope J, Cutler J, et al. Identification of risk factors in hypertensive patients: contribution of randomized controlled trials through an individual patient database. Circulation. 1999 Nov;2(18):e88–94. 10.1161/01.cir.100.18.e88 10545441

[72] Fried LF, Shlipak MG, Crump C, Bleyer AJ, Gottdiener JS, Kronmal RA, et al. Renal insufficiency as a predictor of cardiovascular outcomes and mortality in elderly individuals. J Am Coll Cardiol. 2003 Apr;16(8):1364–72. 10.1016/s0735-1097(03)00163-3 12706933

[73] Henry RMA, Kostense PJ, Bos G, Dekker JM, Nijpels G, Heine RJ, et al. Mild renal insufficiency is associated with increased cardiovascular mortality: the Hoorn Study. Kidney Int. 2002 Oct;62(4):1402–7. 10.1111/j.1523-1755.2002.kid571.x 12234312

[74] Muntner P, He J, Hamm L, Loria C, Whelton PK. Renal insufficiency and subsequent death resulting from cardiovascular disease in the United States. J Am Soc Nephrol. 2002 Mar;13(3):745–53. 10.1681/ASN.V133745 11856780

[75] Wannamethee SG, Shaper AG, Perry IJ. Serum creatinine concentration and risk of cardiovascular disease: a possible marker for increased risk of stroke. Stroke. 1997 Mar;28(3):557–63. 10.1161/01.str.28.3.557 9056611

[76] Culleton BF, Larson MG, Wilson PW, Evans JC, Parfrey PS, Levy D. Cardiovascular disease and mortality in a community-based cohort with mild renal insufficiency. Kidney Int. 1999 Dec;56(6):2214–9. 10.1046/j.1523-1755.1999.00773.x 10594797

[77] Manjunath G, Tighiouart H, Ibrahim H, MacLeod B, Salem DN, Griffith JL, et al. Level of kidney function as a risk factor for atherosclerotic cardiovascular outcomes in the community. J Am Coll Cardiol. 2003 Jan;41(1):47–55. 10.1016/s0735-1097(02)02663-3 12570944

[78] Go AS, Chertow GM, Fan D, McCulloch CE, Hsu C. yuan. Chronic kidney disease and the risks of death, cardiovascular events, and hospitalization. N Engl J Med. 2004 Sep 23;351(13):1296–305. 10.1056/NEJMoa041031 15385656

[79] Foley RN, Murray AM, Li S, Herzog CA, McBean AM, Eggers PW, et al. Chronic kidney disease and the risk for cardiovascular disease, renal replacement, and death in the United States Medicare population, 1998 to 1999. J Am Soc Nephrol. 2005 Feb;16(2):489–95. 10.1681/ASN.2004030203 15590763

[80] Sarnak MJ, Levey AS, Schoolwerth AC, Coresh J, Culleton B, Hamm LL, et al. Kidney disease as a risk factor for development of cardiovascular disease: a statement from the American Heart Association councils on kidney in Cardiovascular Disease, high blood pressure research, clinical cardiology, and Epidemiology and Prevention. Hypertension. 2003 Nov;42(5):1050–65. 10.1161/01.HYP.0000102971.85504.7c 14604997

[81] Vanholder R, Massy Z, Argiles A, Spasovski G, Verbeke F, Lameire N, et al. Chronic kidney disease as cause of cardiovascular morbidity and mortality. Nephrol Dial Transplant. 2005 Jun;20(6):1048–56. 10.1093/ndt/gfh813 15814534

[82] Weiner DE, Tighiouart H, Amin MG, Stark PC, MacLeod B, Griffith JL, et al. Chronic kidney disease as a risk factor for cardiovascular disease and all-cause mortality: a pooled analysis of community-based studies. J Am Soc Nephrol. 2004 May;15(5):1307–15. 10.1097/01.asn.0000123691.46138.e2 15100371

[83] Iseki K, Ikemiya Y, Iseki C, Takishita S. Proteinuria and the risk of developing end-stage renal disease. Kidney Int. 2003 Apr;63(4):1468–74. 10.1046/j.1523-1755.2003.00868.x 12631363

[84] Gansevoort RT, Matsushita K, van der Velde M, Astor BC, Woodward M, Levey AS, et al. Lower estimated GFR and higher albuminuria are associated with adverse kidney outcomes. A collaborative meta-analysis of general and high-risk population cohorts. Kidney Int. 2011 Jul;80(1):93–104. 10.1038/ki.2010.531 PMC3959732 21289597

[85] Matsushita K, van der Velde M, Astor BC, Woodward M, Levey AS, de Jong PE et al. Association of estimated glomerular filtration rate and albuminuria with all-cause and cardiovascular mortality: a collaborative meta-analysis of general population cohorts. Lancet. 2010 Jun 12;375(9731):2073–81. 10.1016/S0140-6736(10)60674-5 PMC3993088 20483451

[86] Astor BC, Matsushita K, Gansevoort RT, van der Velde M, Woodward M, Levey AS, et al. Lower estimated glomerular filtration rate and higher albuminuria are associated with mortality and end-stage renal disease. A collaborative meta-analysis of kidney disease population cohorts. Kidney Int. 2011 Jun;79(12):1331–40. 10.1038/ki.2010.550 PMC3917543 21289598

[87] van der Velde M, Matsushita K, Coresh J, Astor BC, Woodward M, Levey A, et al. Lower estimated glomerular filtration rate and higher albuminuria are associated with all-cause and cardiovascular mortality. A collaborative meta-analysis of high-risk population cohorts. Kidney Int. 2011 Jun;79(12):1341–52. 10.1038/ki.2010.536 21307840

[88] Kido R, Shibagaki Y, Iwadoh K, Nakajima I, Fuchinoue S, Fujita T et al. Very low but stable glomerular filtration rate after living kidney donation: is the concept of “chronic kidney disease” applicable to kidney donors? Clin Exp Nephrol. 2010 Aug;14(4):356–62. 10.1007/s10157-010-0279-y 20339892

[89] Barri YM, Parker T, Daoud Y, Glassock RJ. Definition of chronic kidney disease after uninephrectomy in living donors: what are the implications? Transplantation. 2010 Sep 15;90(5):575–80. 10.1097/TP.0b013e3181e64237 20562736

[90] Chu KH, Poon CKY, Lam CM, Cheuk A, Yim KF, Lee W, et al. Long-term outcomes of living kidney donors: a single centre experience of 29 years. Nephrol (Carlton). 2012 Jan;17(1):85–8. 10.1111/j.1440-1797.2011.01524.x 21919999

[91] Barri Y, Parker T, Kaplan B, Glassock R. Primum non Nocere: is chronic kidney disease staging appropriate in living kidney transplant donors? Am J Transplant. 2009 Apr;9(4):657–60. 10.1111/j.1600-6143.2009.02562.x 19344458

[92] Matas AJ, Ibrahim HN. The unjustified classification of kidney donors as patients with CKD: critique and recommendations. Clin J Am Soc Nephrol. 2013 Aug;8(8):1406–13. 10.2215/CJN.02110213 PMC3731898 23813555

[93] Geddes CC, Wan R. Response to ‘Chronically decreased GFR and cardiovascular risk in living kidney donors.’Kidney International. 2008 Feb2;73(4):509–10. 10.1038/sj.ki.5002665 18235524

[94] Cho HJ, Choi SW, Bae WJ, Kim SJ, Hong SH, Lee JY, et al. Change in renal function following laparoscopic donor nephrectomy using 99 mTc-diethylenetriaminepentaacetic acid scan. World J Urol. 2015 May;33(5):719–23. 10.1007/s00345-014-1408-0 25253655

[95] Lenihan CR, Busque S, Derby G, Blouch K, Myers BD, Tan JC. Longitudinal study of living kidney donor glomerular dynamics after nephrectomy. J Clin Invest. 2015 Mar 2;125(3):1311–8. 10.1172/JCI78885 PMC4362245 25689253

[96] Saran R, Marshall SM, Madsen R, Keavey P, Tapson JS. Long-term follow-up of kidney donors: a longitudinal study. Nephrol Dial Transplant. 1997 Aug;12(8):1615–21. 10.1093/ndt/12.8.1615 9269638

[97] Fehrman-Ekholm I, Kvarnström N, Söfteland JM, Lennerling A, Rizell M, Odén A, et al. Post-nephrectomy development of renal function in living kidney donors: a cross-sectional retrospective study. Nephrol Dial Transplant. 2011 Jul;26(7):2377–81. 10.1093/ndt/gfr161 21459783

[98] Matas AJ, Vock DM, Ibrahim HN. GFR ≤ 25 years postdonation in living kidney donors with (vs. without) a first-degree relative with ESRD. Am J Transplant. 2018 Mar;18(3):625–31. 10.1111/ajt.14525 PMC5820146 28980397

[99] Haugen AJ, Hallan S, Langberg NE, Dahle DO, Pihlstrøm H, Birkeland KI et al. Increased risk of ischaemic heart disease after kidney donation.Nephrology Dialysis Transplantation. 2021 Feb 24;gfab054. 10.1093/ndt/gfab054 PMC9035350 33624826

[100] Reisaeter AV, Røislien J, Henriksen T, Irgens LM, Hartmann A. Pregnancy and birth after kidney donation: the norwegian experience. Am J Transplant. 2009 Apr;9(4):820–4. 10.1111/j.1600-6143.2008.02427.x 18853953

[101] Ibrahim HN, Akkina SK, Leister E, Gillingham K, Cordner G, Guo H (2009). Pregnancy outcomes after kidney donation. Am J Transplant.

[102] Garg AX, Nevis IF, McArthur E, Sontrop JM, Koval JJ, Lam NN et al. Gestational Hypertension and Preeclampsia in Living Kidney Donors.New England Journal of Medicine. 2015 Jan8;372(2):124–33. 10.1056/NEJMoa1408932 PMC4362716 25397608

[103] Janki S, Steyerberg EW, Hofman A, IJzermans JNM (2017). Live kidney donation: are concerns about long-term safety justified?—A methodological review. Eur J Epidemiol.

[104] O’Keeffe LM, Ramond A, Oliver-Williams C, Willeit P, Paige E, Trotter P, et al. Mid- and long-term Health Risks in living kidney donors: a systematic review and Meta-analysis. Ann Intern Med. 2018 Feb;20(4):276–84. 10.7326/M17-1235 29379948

[105] Maggiore U, Budde K, Heemann U, Hilbrands L, Oberbauer R, Oniscu GC et al. Long-term risks of kidney living donation: review and position paper by the ERA-EDTA DESCARTES working group. Nephrol Dial Transplant. 2017 Feb 1;32(2):216–23. 10.1093/ndt/gfw429 28186535

[106] Gaston RS, Kumar V, Matas AJ. Reassessing medical risk in living kidney donors. J Am Soc Nephrol. 2015 May;26(5):1017–9. 10.1681/ASN.2014030227 PMC4413760 25255922

[107] Gill JS, Tonelli M. Understanding rare adverse outcomes following living kidney donation.JAMA. 2014 Feb12;311(6):577–9. 10.1001/jama.2013.285142 24519296

[108] Kasiske BL. Outcomes after living kidney donation: what we still need to know and why. Am J Kidney Dis. 2014 Sep;64(3):335–7. 10.1053/j.ajkd.2014.04.013 24797521

[109] Lam NN, Lentine KL, Garg AX. End-stage renal disease risk in live kidney donors: what have we learned from two recent studies? Curr Opin Nephrol Hypertens. 2014 Nov;23(6):592–6. 10.1097/MNH.0000000000000063 PMC4189686 25160076

[110] Matas AJ, Transplantation. Increased ESRD and mortality risk for kidney donors? Nat Rev Nephrol. 2014 Mar;10(3):130–1. 10.1038/nrneph.2014.2 24445743

[111] Kaplan B, Ilahe A. Quantifying risk of kidney donation: the truth is not out there (yet). Am J Transplant. 2014 Aug;14(8):1715–6. 10.1111/ajt.12804 24866857

[112] Boudville N, Garg AX. End-stage renal disease in living kidney donors. Kidney Int. 2014 Jul;86(1):20–2. 10.1038/ki.2013.560 24978379

[113] Poggio ED, Reese PP. The Quest to define individual risk after living kidney donation. Ann Intern Med. 2018 Feb;20(4):296–7. 10.7326/M17-3249 29379960

[114] Lam NN, Lentine KL, Levey AS, Kasiske BL, Garg AX. Long-term medical risks to the living kidney donor. Nat Rev Nephrol. 2015 Jul;11(7):411–9. 10.1038/nrneph.2015.58 25941060

[115] Gill JS. New evidence of the need for living kidney donor follow-up. Am J Transplant. 2018 May;18(5):1041–2. 10.1111/ajt.14716 29498805

[116] Lei HH, Perneger TV, Klag MJ, Whelton PK, Coresh J. Familial aggregation of renal disease in a population-based case-control study. J Am Soc Nephrol. 1998 Jul;9(7):1270–6. 10.1681/ASN.V971270 9644638

[117] O’Dea DF, Murphy SW, Hefferton D, Parfrey PS. Higher risk for renal failure in first-degree relatives of white patients with end-stage renal disease: a population-based study. Am J Kidney Dis. 1998 Nov;32(5):794–801. 10.1016/s0272-6386(98)70135-0 9820449

[118] Skrunes R, Svarstad E, Reisæter AV, Vikse BE. Familial clustering of ESRD in the norwegian population. Clin J Am Soc Nephrol. 2014 Oct;7(10):1692–700. 10.2215/CJN.01680214 PMC4186510 25092600

[119] Freedman BI, Tuttle AB, Spray BJ. Familial predisposition to nephropathy in African-Americans with non-insulin-dependent diabetes mellitus. Am J Kidney Dis. 1995 May;25(5):710–3. 10.1016/0272-6386(95)90546-4 7747724

[120] Ferguson R, Grim CE, Opgenorth TJ (1988). A familial risk of chronic renal failure among blacks on dialysis?. J Clin Epidemiol.

[121] Freedman BI, Spray BJ, Tuttle AB, Buckalew VM. The familial risk of end-stage renal disease in African Americans. Am J Kidney Dis. 1993 Apr;21(4):387–93. 10.1016/s0272-6386(12)80266-6 8465818

[122] Bergman S, Key BO, Kirk KA, Warnock DG, Rostant SG. Kidney disease in the first-degree relatives of African-Americans with hypertensive end-stage renal disease. Am J Kidney Dis. 1996 Mar;27(3):341–6. 10.1016/s0272-6386(96)90356-x 8604702

[123] Wainright JL, Robinson AM, Wilk AR, Klassen DK, Cherikh WS, Stewart DE. Risk of ESRD in prior living kidney donors. Am J Transplant. 2018 May;18(5):1129–39. 10.1111/ajt.14678 29392849

[124] Davis S, Dylewski J, Shah PB, Holmen J, You Z, Chonchol M, et al. Risk of adverse maternal and fetal outcomes during pregnancy in living kidney donors: a matched cohort study. Clin Transpl. 2019 Jan;33(1):e13453. 10.1111/ctr.13453 PMC6342653 30472740

[125] Yoo KD, Lee H, Kim Y, Park S, Park JS, Hong JS, et al. Maternal and fetal outcomes of pregnancies in kidney donors: a 30-year comparative analysis of matched non-donors in a single center. Kidney Res Clin Pract. 2018 Dec;37(4):356–65. 10.23876/j.krcp.18.0050 PMC6312783 30619691

[126] Steiner RW. Normal for now” or “at future risk”: a double standard for selecting young and older living kidney donors. Am J Transplant. 2010 Apr;10(4):737–41. 10.1111/j.1600-6143.2010.03023.x 20199512

[127] Steiner RW. Moving closer to understanding the risks of living kidney donation. Clin Transpl. 2016 Jan;30(1):10–6. 10.1111/ctr.12652 26689427

[128] Steiner RW. You can’t get there from here”: critical obstacles to current estimates of the ESRD risks of young living kidney donors. Am J Transplant. 2019 Jan;19(1):32–6. 10.1111/ajt.15089 30137698

[129] National data - OPTN [Internet]. [cited 2023 Jan 25]. Available from: https://optn.transplant.hrsa.gov/data/view-data-reports/national-data/

[130] Matas AJ, Berglund DM, Vock DM, Ibrahim HN. Causes and timing of end-stage renal disease after living kidney donation. Am J Transplant. 2018 May;18(5):1140–50. 10.1111/ajt.14671 29369517

[131] Anjum S, Muzaale AD, Massie AB, Bae S, Luo X, Grams ME, et al. Patterns of end-stage renal disease caused by diabetes, hypertension, and glomerulonephritis in live kidney donors. Am J Transplant. 2016 Dec;16(12):3540–7. 10.1111/ajt.13917 PMC6116527 27287605

[132] U.S. Department of Health and Human Services Advisory Committee on Organ Transplantation (ACOT). ACOT Recommendations. [Internet]. 2011. Available from: http://organdonor.gov/legislation/advisory.html

[133] Public Law 108–216: Organ Donation and Recovery Improvement Act (ODRIA). Text from: United States Public Laws [Internet]. 2004. Available from: http://www.livingdonorassistance.org/documents/Public%20Law%20108-216_Organ%20Donation%20Act.pdf

[134] Congressional Committee testimony by Dr. James Burdick, Director, Division of Transplantation, Health Resources and Services Administration, U.S. Department of Health and Human Services, reference to ODRIA. Mechanisms to evaluate the long-term effects of living organ donation. [Internet]. 2007. Available from: http://www.hhs.gov/asl/testify/2007/09/t20070925a.htm

[135] U.S. Department of Health and Human Services. Living donor excerpt. [Internet]. 2011. Available from: http://www.organdonor.gov/about/livedonation.html

[136] Living Kidney Donor Follow-Up Conference Writing Group, Leichtman A, Abecassis M, Barr M, Charlton M, Cohen D et al. Living kidney donor follow-up: state-of-the-art and future directions, conference summary and recommendations. Am J Transplant. 2011 Dec;11(12):2561–8. 10.1111/j.1600-6143.2011.03816.x 22054039

[137] LaPointe Rudow D, Hays R, Baliga P, Cohen DJ, Cooper M, Danovitch GM et al. Consensus conference on best practices in live kidney donation: recommendations to optimize education, access, and care. Am J Transplant. 2015 Apr;15(4):914–22. 10.1111/ajt.13173 PMC4516059 25648884

[138] Shelton DL. Kidney donors speak out on risks. Chicago Tribune [Internet]. 2011 [cited 2022 Nov 4]; Available from: https://www.chicagotribune.com/lifestyles/health/ct-kidney-donors-speak-out-on-risks-story.html

[139] Neergaard L. Push to better track living kidney donors’ long-term health. The Denver Post [Internet]. 2018 Jan 30 [cited 2022 Nov 4]; Available from: https://www.denverpost.com/2018/01/29/push-to-better-track-living-kidney-donors-long-term-health/

[140] Hanson CS, Chapman JR, Gill JS, Kanellis J, Wong G, Craig JC et al. Identifying Outcomes that Are Important to Living Kidney Donors: A Nominal Group Technique Study.Clin J Am Soc Nephrol. 2018 Jun7;13(6):916–26. 10.2215/CJN.13441217 PMC5989678 29853616

[141] Ruck JM, Van Pilsum Rasmussen SE, Henderson ML, Massie AB, Segev DL. Interviews of living kidney donors to assess donation-related concerns and information-gathering practices. BMC Nephrol. 2018 Jun 8;19(1):130. 10.1186/s12882-018-0935-0 PMC5994029 29884126

[142] Melton LJ. History of the Rochester Epidemiology Project. Mayo Clin Proc. 1996 Mar;71(3):266–74. 10.4065/71.3.266 8594285

[143] St Sauver JL, Grossardt BR, Yawn BP, Melton LJ, Pankratz JJ, Brue SM, et al. Data resource profile: the Rochester Epidemiology Project (REP) medical records-linkage system. Int J Epidemiol. 2012 Dec;41(6):1614–24. 10.1093/ije/dys195 PMC3535751 23159830

[144] St. Sauver JL, Grossardt BR, Yawn BP, Melton LJ, Rocca WA. Use of a Medical Records Linkage System to Enumerate a Dynamic Population Over Time: The Rochester Epidemiology Project. Am J Epidemiol. 2011 May 1;173(9):1059–68. 10.1093/aje/kwq482 PMC3105274 21430193

[145] Rocca WA, Yawn BP, St Sauver JL, Grossardt BR, Melton LJ. History of the Rochester Epidemiology Project: half a century of medical records linkage in a US population. Mayo Clin Proc. 2012 Dec;87(12):1202–13. 10.1016/j.mayocp.2012.08.012 PMC3541925 23199802

[146] St Sauver JL, Grossardt BR, Leibson CL, Yawn BP, Melton LJ, Rocca WA. Generalizability of epidemiological findings and public health decisions: an illustration from the Rochester Epidemiology Project. Mayo Clin Proc. 2012 Feb;87(2):151–60. 10.1016/j.mayocp.2011.11.009 PMC3538404 22305027

[147] Rule AD, Bergstralh EJ, Melton LJ, Li X, Weaver AL, Lieske JC. Kidney stones and the risk for chronic kidney disease. Clin J Am Soc Nephrol. 2009 Apr;4(4):804–11. 10.2215/CJN.05811108 PMC2666438 19339425

[148] Pimentel SD, Large (2016). Sparse optimal matching with R package rebalance. Observational Stud.

[149] Pimentel SD, Kelz RR, Silber JH, Rosenbaum PR. Large, Sparse Optimal Matching With Refined Covariate Balance in an Observational Study of the Health Outcomes Produced by New Surgeons. Journal of the American Statistical Association. 2015 Apr 3;110(510):515–27. 10.1080/01621459.2014.997879 PMC4531000 26273117

[150] Engels EA, Fraser GE, Kasiske BL, Snyder JJ, Utt J, Lynch CF, et al. Cancer risk in living kidney donors. Am J Transplant. 2022 Aug;22(8):2006–15. 10.1111/ajt.17082 PMC9357116 35510728

[151] Shi X, Miao W, Tchetgen ET. A selective review of negative control methods in Epidemiology. Curr Epidemiol Rep. 2020 Dec;7(1):190–202. 10.1007/s40471-020-00243-4 PMC8118596 33996381

[152] Matas AJ, Hays RE, Ibrahim HN. A Case-Based Analysis of Whether Living Related Donors Listed for Transplant Share ESRD Causes with Their Recipients. Clin J Am Soc Nephrol. 2017 Apr 3;12(4):663–8. 10.2215/CJN.11421116 PMC5383394 28249957

[153] Henderson ML, Thomas AG, Shaffer A, Massie AB, Luo X, Holscher CM, et al. The National Landscape of living kidney Donor Follow-Up in the United States. Am J Transplant. 2017 Dec;17(12):3131–40. 10.1111/ajt.14356 PMC5690895 28510355

[154] Rodrigue JR, Fleishman A, Sokas CM, Schold JD, Morrissey P, Whiting J et al. Rates of Living Kidney Donor Follow-up: Findings From the KDOC Study. Transplantation. 2019 Jul;103(7):e209–10. 10.1097/TP.0000000000002721 PMC6597183 31241558

[155] Reed RD, MacLennan PA, Shelton BA, Mustian MN, Blackburn J, Smith SC et al. Center Variation and Risk Factors for Failure to Complete Six-Month Post-Donation Follow-up among Obese Living Kidney Donors. Transplantation. 2019 Jul;103(7):1450–6. 10.1097/TP.0000000000002508 PMC6597173 31241556

