# Supplementary material for: The Minnesota attributable risk of kidney donation (MARKD) study: a retrospective cohort study of long-term (> 50 year) outcomes after kidney donation compared to well-matched healthy controls
Source: BMC Nephrol. 2023 May 1;24:121. doi: 10.1186/s12882-023-03149-7 (PMC10152793; doi:10.1186/s12882-023-03149-7)
Supplement: Supplementary file 1 — Supplementary Material 1 [file 12882_2023_3149_MOESM1_ESM.pdf]

Appendix 1: Exclusionary Conditions Identified by ICD-9, ICD-10, HICDA and Berkson Codes

Accident Cerebrovascular
Amaurosis Fugax
Amyloidosis
Anemia Hemolytic
Aneurysm Aortic
Aneurysm Cerebral
Angina
Arrest Cardiac
Arrhythmia Cardiac
Asbestosis
Ascites
Ataxia Telangiectasia
Attack Transient Ischemic
Bronchitis Chronic
Carcinoid Tumor Malignant
Carcinoma Merkel Cell
Cardiomyopathy
Cholangitis Sclerosing
Cholestasis
Cirrhosis Liver
Cirrhosis Primary Billiary
Colitis Ischemic
Colitis Ulcerative CUC
Deficiency Alpha1 Antitrypsin
Dementia
Depression Always
Diabetes Mellitus comps
Dialysis
Disease Addisons
Disease Autoimmune
Disease Cerebrovascular
Disease Chronic Obstructive Pulmonary
Disease Cold Agglutin
Disease Connective Tissue
Disease Coronary Artery
Disease Crohns
Disease Cushings
Disease Graft VS Host
Disease Heart Congenital Always
Disease Heart Ischemic ACUTE
Disease Inflammatory Bowel Always
Disease Kidney Chronic
Disease Liver Alcoholic Always
Disease Lung Interstitial
Disease Parkinsons

Disease Peripheral Vascular
Disease Renal Always
Disease Sickle Cell
Disease Wilson
Disorder Autism Spectrum
Disorder Bipolar
Disorder Bleeding
Disorder Immunodeficiency
Donor Kidney status
Dysfunction Left Ventricular
Dysplasia Fibromuscular
Emphysema
Encephalopathy Hepatic
Endocarditis
Ependymoma
Epidermolysis Bullosa
Failure Heart
Failure Hepatic
Failure Renal AKI
Failure Respiratory
Fibrillation Atrial
Fibrosis Pulmonary
Glomerulonephritis
Granulomatosis Wegeners
HIV AIDS
Hematuria Always
Hemochromatosis
Hepatitis Autoimmune
Hepatitis B
Hepatitis C Always
Hepatitis
Histoplasmosis
Hypercalcemia
Hypertension Intracranial
Hypertension Pulmonary
Infarction Myocardial
Infarction Pulmonary
Injury Spinal Cord
Insufficiency Renal
Leukemia
Lupus Erythematosus Systemic
Malabsorption
Malformation Chiari
Malignancy Hematologic
Melanoma
Myeloma Multiple MGUS
Myopathy
Neoplasm Malignant Bladder

Neoplasm Malignant Bone Any Site
Neoplasm Malignant Brain
Neoplasm Malignant Breast
Neoplasm Malignant Cervix Female
Neoplasm Malignant Endometrium
Neoplasm Malignant Endometrium Uterus
Neoplasm Malignant Esophagus
Neoplasm Malignant Gallbladder Extrahepatic Bile Ducts
Neoplasm Malignant Genital Organs Female
Neoplasm Malignant Hypopharynx
Neoplasm Malignant Intestine Large Colorectal
Neoplasm Malignant Intestine Small
Neoplasm Malignant Kidney
Neoplasm Malignant Larynx
Neoplasm Malignant Lip Oral Pharynx
Neoplasm Malignant Liver Intrahepatic Bile Ducts
Neoplasm Malignant Lung Bronchus Trachea
Neoplasm Malignant Nasopharynx
Neoplasm Malignant Oropharynx
Neoplasm Malignant Ovary
Neoplasm Malignant Pancreas
Neoplasm Malignant Penis
Neoplasm Malignant Prostate
Neoplasm Malignant Stomach
Neoplasm Malignant Thyroid
Neoplasm Malignant Urinary System
Osteogenesis Imperfecta
Paralysis
Pemphigus Pemphigoid
Peritonitis Spontaneous Bacterial
Phenomenon Raynauds
Polyangiitis
Polycystic Kidney
Proteinuria Pregnancy Related
Purpura Idiopathic Thrombocytic
Retardation Mental Always
Sarcoidosis
Sarcoma Kaposis
Schizophrenia
Scleroderma Circumscribed
Scleroderma Systemic
Sclerosis Multiple
Silicosis
Status Coronary Artery Vascular Procedures
Status PTCA
Status Pacemaker Cardiac Defibrillator
Status Prosthetic Heart Valve including complications
Status Skin Graft

Status Transplant Heart
Status Transplant Kidney
Status Transplant Liver
Stenosis Heart Valve
Stenosis Renal Artery
Stroke Hemorrhagic
Stroke Ischemic
Stroke Sequelae
Suicide Gestures
Syndrome Angelman
Syndrome Antiphospholipid
Syndrome Churg Strauss
Syndrome Cri Du Chat
Syndrome Downs
Syndrome Ehlers Danlos
Syndrome Fetal Alcohol
Syndrome FragileX
Syndrome Goodpastures
Syndrome Guillaine Barre
Syndrome HELLP
Syndrome Hepatorenal
Syndrome Hypoplastic Left Heart
Syndrome Marfan
Syndrome Polyendocrine
Syndrome Prader Willi
Thrombosis Portal Vein
Thrombosis superficial deep vein
Trisomy 13
Trisomy 18
Tumor Brain
Varix Esophageal
Vasculitis
