# Supplementary material for: The Minnesota attributable risk of kidney donation (MARKD) study: a retrospective cohort study of long-term (> 50 year) outcomes after kidney donation compared to well-matched healthy controls
Source: BMC Nephrol. 2023 May 1;24:121. doi: 10.1186/s12882-023-03149-7 (PMC10152793; doi:10.1186/s12882-023-03149-7)
Supplement: Supplementary file 2 — Supplementary Material 2 [file 12882_2023_3149_MOESM2_ESM.pdf]

Appendix 2: Study survey

Kidney Health Survey

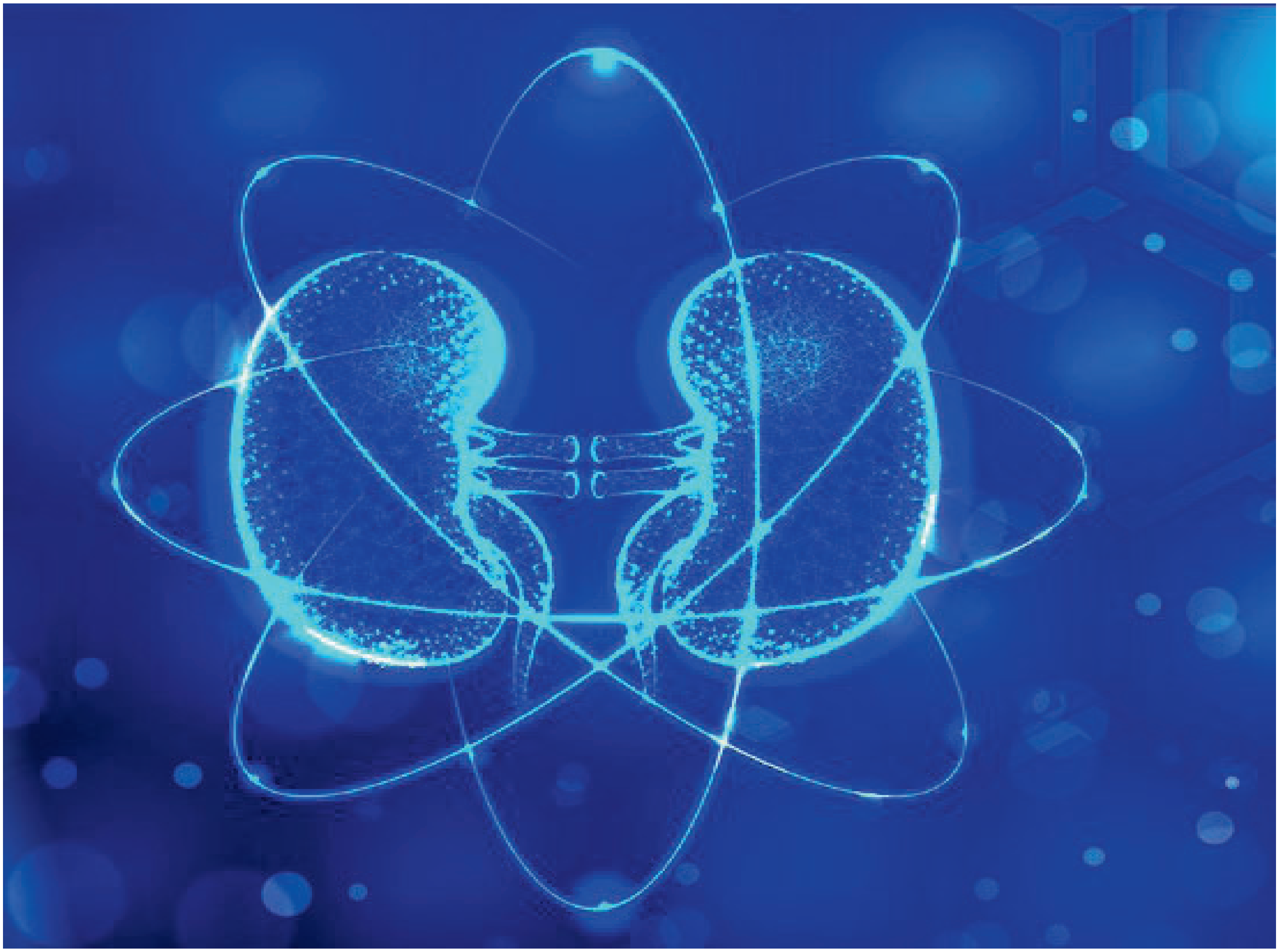

Survey Research Center

1-8

REP ID
Name
Address

9-16 Date: <date>

Please correct above any of your contact information that is incorrect on the label.
We will be asking you if events occurred before or after the date on the label above.

Additional contact information in case we have further research questions. (Optional)

17-26 Best phone number to reach me is: (__ __ __) - __ __ __ - __ __ __ __

27 Email address: ____

28-35 **1. Today's Date:** __ __/__ __/__ __ __ __
Month Day Year

INSTRUCTIONS:

This survey asks about your health. Please answer each question thinking about yourself. Please answer all questions to the best of your ability. If you do not know the exact year for a question but can make a reasonable guess, that would be acceptable. Some questions will ask whether events happened before or after the date on the label above.

Use either a ballpoint pen or pencil to complete.

Answer questions by marking an "X" in the box that best describes your answer or filling in the blanks as directed.

If you make a mistake or change your mind, clearly erase the mark or indicate the change before marking a new answer.

If you are not sure about how to answer a question, please give the best answer you can and make a comment in the margin.

Medical Questions

36 **2. Has a doctor or other health care professional <u>ever</u> told you that you have or had kidney disease (not including kidney stones) or abnormal kidney function?**

1 ☐ Yes 2 ☐ No

37-40 **If yes, what year were you first told:** __ __ __ __ Year

Please explain how it is being treated:

41 ______________________________

3. Did a doctor or other health care professional <u>ever</u> tell you that you have or had any of the following health conditions? (Check one box on each line.)

		Yes	No	Year first diagnosed with health condition
42<br>43-46	**Blood in your urine (hematuria), lasting 6 or more months, not including during pregnancy**	1 ☐	2 ☐	__ __ __ __ Year
47<br>48-51	**Protein in your urine (proteinuria) that was high or abnormal, lasting 6 or more months, not including during pregnancy**	1 ☐	2 ☐	__ __ __ __ Year
52<br>53-56	**High cholesterol treated with medication**	1 ☐	2 ☐	__ __ __ __ Year
57<br>58-61	**Hypertension (high blood pressure) treated with medication**	1 ☐	2 ☐	__ __ __ __ Year
62<br>63-66	**Heart failure (congestive heart disease)**	1 ☐	2 ☐	__ __ __ __ Year
67<br>68-71	**Heart attack (myocardial infarction)**	1 ☐	2 ☐	__ __ __ __ Year
72<br>73-76	**Stroke (cerebrovascular accident (CVA) or transient ischemic attack (TIA))**	1 ☐	2 ☐	__ __ __ __ Year

Continues on next page...

Did a doctor or other health care professional <u>ever</u> tell you that you have or had any of the following health conditions?

Coronary artery disease or angina (chest pain)

77 1 ☐ Yes 2 ☐ No

78-81 **If yes, first year diagnosed with health condition:** __ __ __ __ Year

Treatment:

82 0 ☐ None 1 ☐ Medication

Diabetes

83 1 ☐ Yes 2 ☐ No

84-87 **If yes, first year diagnosed with health condition:** __ __ __ __ Year

Treatment: (Check all that apply.)

88-91 1 ☐ Diet 1 ☐ Insulin 1 ☐ Pill 1 ☐ No treatment

4. **Have you ever had any of the following medical procedures or surgeries? If you had the procedure/surgery more than once, list the first year.** (Check one box on each line.)

		Yes	No	Year of procedure or surgery
92<br>93-96	**<u>Coronary balloon angioplasty or stenting</u> (using a balloon or metal stent to open a blood vessel to your heart)**	1 ☐	2 ☐	__ __ __ __ Year
97<br>98-101	**<u>Heart bypass surgery</u>**	1 ☐	2 ☐	__ __ __ __ Year
102<br>103-106	**An <u>operation to unclog or bypass the arteries in your leg</u>**	1 ☐	2 ☐	__ __ __ __ Year

5. **Have you ever been diagnosed with a cancer?** (Do not include basal or squamous cell skin cancer.)

107 1 ☐ Yes 2 ☐ No

If yes, please indicate the type(s) of cancer and the year in which it was diagnosed.

108 **Type of Cancer**	109-112 **Year FIRST Diagnosed**
____________________	__ __ __ __ Year
____________________	__ __ __ __ Year
____________________	__ __ __ __ Year

Physical Tests

6. **For each of the following tests, please record your most recent test results below.**

	Most Recent Result	Date Done	
**Height:**	____Feet __ __ Inches	__ __/__ __/__ __ __ __<br>Month Day Year	1 ☐ Don't know
**Weight:**	__ __ __ Pounds	__ __/__ __/__ __ __ __<br>Month Day Year	1 ☐ Don't know
**Blood Pressure:**	__ __ __/__ __ __mmHg	__ __/__ __/__ __ __ __<br>Month Day Year	1 ☐ Don't know
**Blood test – serum creatinine:**	__.__ __ mg/dL	__ __/__ __/__ __ __ __<br>Month Day Year	1 ☐ Don't know

For the following questions, please refer to the reference date on the label found on page 1 of this survey.

Family History of Kidney Disease, Diabetes, and Hypertension

7. Do or did any of your blood relatives have abnormal kidney function or kidney disease (not including kidney stones)?

1 ☐ Yes   2 ☐ No   3 ☐ Don't know

For each blood relative with *kidney disease*, please answer the following 4 questions:

a) Are they a close relative (parent, brother or sister [full or half], or child) or a more distant relative (cousin, grandparent, aunt, uncle, niece, or nephew)?

b) Did they get diagnosed with kidney disease that was likely before or likely after the date on the label on page 1?

c) If they developed kidney failure (kidneys stopped working requiring dialysis or a kidney transplant to survive), did this occur likely before or likely after the date on the label on page 1?

d) If known, write in the cause of their kidney disease.

First blood relative with kidney disease

a) Is this a:
1 ☐ Close blood relative
2 ☐ Distant blood relative

b) Was the kidney disease diagnosed:
1 ☐ Before the date on the label
2 ☐ After the date on the label

c) Did they develop kidney failure?
1 ☐ Yes, before the date on the label
2 ☐ Yes, after the date on the label
3 ☐ No

d) If known, what was the cause of their kidney disease: ____________________

Continues next page...

Second blood relative with kidney disease

a) Is this a:
1 ☐ Close blood relative
2 ☐ Distant blood relative

b) Was the kidney disease diagnosed:
1 ☐ Before the date on the label
2 ☐ After the date on the label

c) Did they develop kidney failure?
1 ☐ Yes, before the date on the label
2 ☐ Yes, after the date on the label
3 ☐ No

d) If known, what was the cause of their kidney disease: ____________________________

Third blood relative with kidney disease

a) Is this a:
1 ☐ Close blood relative
2 ☐ Distant blood relative

b) Was the kidney disease diagnosed:
1 ☐ Before the date on the label
2 ☐ After the date on the label

c) Did they develop kidney failure?
1 ☐ Yes, before the date on the label
2 ☐ Yes, after the date on the label
3 ☐ No

d) If known, what was the cause of their kidney disease: ____________________________

Fourth blood relative with kidney disease

a) Is this a:
1 ☐ Close blood relative
2 ☐ Distant blood relative

b) Was the kidney disease diagnosed:
1 ☐ Before the date on the label
2 ☐ After the date on the label

c) Did they develop kidney failure?
1 ☐ Yes, before the date on the label
2 ☐ Yes, after the date on the label
3 ☐ No

d) If known, what was the cause of their kidney disease: ____________________________

8. **Do or did any close <u>blood</u> relatives (parents, brothers, sisters, children) have <u>diabetes</u>?**

1 ☐ Yes   2 ☐ No   3 ☐ Don't know

For each close blood relative with <u>diabetes</u>, answer the following questions:

Blood relative	What is their relationship to you?		Did diabetes begin before or after the date on the label?	Is their diabetes Type 1, Type 2, or unknown?
**#1**	1 ☐ Son<br>2 ☐ Daughter<br>3 ☐ Mother	4 ☐ Father<br>5 ☐ Brother/Sister (full or half)	1 ☐ Before<br>2 ☐ After<br>3 ☐ Unknown	1 ☐ Type 1<br>2 ☐ Type 2<br>3 ☐ Unknown
**#2**	1 ☐ Son<br>2 ☐ Daughter<br>3 ☐ Mother	4 ☐ Father<br>5 ☐ Brother/Sister (full or half)	1 ☐ Before<br>2 ☐ After<br>3 ☐ Unknown	1 ☐ Type 1<br>2 ☐ Type 2<br>3 ☐ Unknown
**#3**	1 ☐ Son<br>2 ☐ Daughter<br>3 ☐ Mother	4 ☐ Father<br>5 ☐ Brother/Sister (full or half)	1 ☐ Before<br>2 ☐ After<br>3 ☐ Unknown	1 ☐ Type 1<br>2 ☐ Type 2<br>3 ☐ Unknown
**#4**	1 ☐ Son<br>2 ☐ Daughter<br>3 ☐ Mother	4 ☐ Father<br>5 ☐ Brother/Sister (full or half)	1 ☐ Before<br>2 ☐ After<br>3 ☐ Unknown	1 ☐ Type 1<br>2 ☐ Type 2<br>3 ☐ Unknown

9. **Do or did any close blood relatives (parents, brothers, sisters, children) have <u>hypertension</u> (high blood pressure)?**

1 ☐ Yes   2 ☐ No   3 ☐ Don't know

For each close blood relative with <u>hypertension</u>, answer the following questions:

Blood relative	What is their relationship to you?			Did hypertension begin before or after the date on the label?
**#1**	1 ☐ Son<br>2 ☐ Daughter	3 ☐ Mother<br>4 ☐ Father	5 ☐ Brother/Sister (full or half)	1 ☐ Before<br>2 ☐ After<br>3 ☐ Unknown
**#2**	1 ☐ Son<br>2 ☐ Daughter	3 ☐ Mother<br>4 ☐ Father	5 ☐ Brother/Sister (full or half)	1 ☐ Before<br>2 ☐ After<br>3 ☐ Unknown
**#3**	1 ☐ Son<br>2 ☐ Daughter	3 ☐ Mother<br>4 ☐ Father	5 ☐ Brother/Sister (full or half)	1 ☐ Before<br>2 ☐ After<br>3 ☐ Unknown
**#4**	1 ☐ Son<br>2 ☐ Daughter	3 ☐ Mother<br>4 ☐ Father	5 ☐ Brother/Sister (full or half)	1 ☐ Before<br>2 ☐ After<br>3 ☐ Unknown

10. Have you smoked at least 100 cigarettes in your life?

1 ☐ Yes   2 ☐ No   3 ☐ Don't know

Which choice below describes your current cigarette smoking activity? (Check best choice.)

1 ☐ Every day   2 ☐ Some days   3 ☐ Used to smoke but currently do not

Year started: __ __ __ __

Year stopped (if applicable): __ __ __ __

WOMEN ONLY: Pregnancies (Men skip to question 12.)

11. How many times have you been pregnant?

__ __ Number of pregnancies   1 ☐ Never been pregnant, skip to question 12

For each pregnancy (oldest to youngest), answer the following questions.

**Pregnancy end date or birth date (year only)?**	**Pregnancy outcome?**	**Gestational length?**	**Pregnancy problems?** (Mark all that apply.)
__ __ __ __ Year	1 ☐ Single birth	1 ☐ Full-term (greater than or equal to 37 weeks)	1 ☐ Hypertension
	2 ☐ Twins	2 ☐ Pre-term (less than 37 weeks)	1 ☐ Gestational diabetes
	3 ☐ Triplets	3 ☐ Unknown	1 ☐ Protein in urine
	4 ☐ 4 or more		1 ☐ Eclampsia
	5 ☐ Abortion		1 ☐ Preeclampsia
	6 ☐ Miscarriage (less than 20 weeks)		1 ☐ Other, specify: ____________
	7 ☐ Fetal death (greater than 20 weeks)		

Continues on next page...

For each pregnancy (oldest to youngest), answer the following questions.

**Pregnancy end date or birth date (year only)?**	**Pregnancy outcome?**	**Gestational length?**	**Pregnancy problems?** (Mark all that apply.)
__ __ __ __<br>Year	1 ☐ Single birth<br>2 ☐ Twins<br>3 ☐ Triplets<br>4 ☐ 4 or more<br>5 ☐ Abortion<br>6 ☐ Miscarriage (less than 20 weeks)<br>7 ☐ Fetal death (greater than 20 weeks)	1 ☐ Full-term (greater than or equal to 37 weeks)<br>2 ☐ Pre-term (less than 37 weeks)<br>3 ☐ Unknown	1 ☐ Hypertension<br>1 ☐ Gestational diabetes<br>1 ☐ Protein in urine<br>1 ☐ Eclampsia<br>1 ☐ Preeclampsia<br>1 ☐ Other, specify: ________
__ __ __ __<br>Year	1 ☐ Single birth<br>2 ☐ Twins<br>3 ☐ Triplets<br>4 ☐ 4 or more<br>5 ☐ Abortion<br>6 ☐ Miscarriage (less than 20 weeks)<br>7 ☐ Fetal death (greater than 20 weeks)	1 ☐ Full-term (greater than or equal to 37 weeks)<br>2 ☐ Pre-term (less than 37 weeks)<br>3 ☐ Unknown	1 ☐ Hypertension<br>1 ☐ Gestational diabetes<br>1 ☐ Protein in urine<br>1 ☐ Eclampsia<br>1 ☐ Preeclampsia<br>1 ☐ Other, specify: ________
__ __ __ __<br>Year	1 ☐ Single birth<br>2 ☐ Twins<br>3 ☐ Triplets<br>4 ☐ 4 or more<br>5 ☐ Abortion<br>6 ☐ Miscarriage (less than 20 weeks)<br>7 ☐ Fetal death (greater than 20 weeks)	1 ☐ Full-term (greater than or equal to 37 weeks)<br>2 ☐ Pre-term (less than 37 weeks)<br>3 ☐ Unknown	1 ☐ Hypertension<br>1 ☐ Gestational diabetes<br>1 ☐ Protein in urine<br>1 ☐ Eclampsia<br>1 ☐ Preeclampsia<br>1 ☐ Other, specify: ________

Continues on next page...

For each pregnancy, answer the following questions.

**Pregnancy end date or birth date (year only)?**	**Pregnancy outcome?**	**Gestational length?**	**Pregnancy problems?** (Mark all that apply.)
____ Year	1 ☐ Single birth<br>2 ☐ Twins<br>3 ☐ Triplets<br>4 ☐ 4 or more<br>5 ☐ Abortion<br>6 ☐ Miscarriage (less than 20 weeks)<br>7 ☐ Fetal death (greater than 20 weeks)	1 ☐ Full-term (greater than or equal to 37 weeks)<br>2 ☐ Pre-term (less than 37 weeks)<br>3 ☐ Unknown	1 ☐ Hypertension<br>1 ☐ Gestational diabetes<br>1 ☐ Protein in urine<br>1 ☐ Eclampsia<br>1 ☐ Preeclampsia<br>1 ☐ Other, specify: ________

12. Have you had any other major health problems that started before the date on the label?

1 ☐ Yes 2 ☐ No

If yes, please describe:

____

____

____

____

13. Have you had any other major health problems that started after the date on the label?

1 ☐ Yes 2 ☐ No

If yes, please describe:

____

____

____

____

Thank you for your time in completing this survey!
